# Microglial dynamics associate with the spatial pattern of tau deposition in early Alzheimer’s disease

**DOI:** 10.1186/s13195-026-02230-1

**Published:** 2026-10-12

**Authors:** Marco Öchsner, Matthias Brendel, Nicolai Franzmeier, Lena Trappmann, Mirlind Zaganjori, Ersin Ersoezlue, Carolin Kurz, Jan Haeckert, Julia Utecht, Philine Marie Schirge, Boris Papazov, Oliver Pogarell, Daniel Janowitz, Katharina Buerger, Michael Ewers, Carla Palleis, Endy Weidinger, Gloria Biechele, Sebastian Schuster, Anika Finze, Florian Eckenweber, Rainer Rupprecht, Axel Rominger, Oliver Goldhardt, Timo Grimmer, Daniel Keeser, Sophia Stoecklein, Olaf Dietrich, Peter Bartenstein, Johannes Levin, Günter Höglinger, Robert Perneczky, Boris-Stephan Rauchmann

**Affiliations:** 1 Department of Neuroradiology, LMU University Hospital, LMU Munich, Marchioninistraße 15, Munich, 81377 Germany; 2 Department of Nuclear Medicine, LMU University Hospital, LMU Munich, Munich, Germany; 3 Munich Cluster for Systems Neurology (SyNergy), Munich, Germany; 4 Institute for Stroke and Dementia Research (ISD), LMU University Hospital, LMU Munich, Munich, Germany; 5 Institute of Neuroscience and Physiology, Department of Psychiatry and Neurochemistry, University of Gothenburg, The Sahlgrenska Academy , Mölndal and Gothenburg, Sweden; 6 Department of Psychiatry and Psychotherapy, LMU University Hospital, LMU Munich, Munich, Germany; 7 Department of Psychiatry, Psychotherapy, and Psychosomatics, University of Augsburg, Augsburg, Germany; 8 Department of Radiology, LMU University Hospital, LMU Munich, Munich, Germany; 9 German Center for Neurodegenerative Diseases, Munich, Germany; 10 Department of Neurology, LMU University Hospital, LMU Munich, Munich, Germany; 11 Department of Psychiatry and Psychotherapy, University of Regensburg, Regensburg, Germany; 12 Department of Nuclear Medicine, University of Bern, Inselspital, Bern, Switzerland; 13 Department of Neurology, School of Medicine, TUM University Hospital, Technical University of Munich, Munich, Germany; 14 Department of Neurology, Hannover Medical School, Hannover, Germany; 15 Aging Epidemiology Research Unit, School of Public Health, Imperial College London, London, United Kingdom; 16 Shefﬁeld Institute for Translational Neuroscience, University of Shefﬁeld, Shefﬁeld, United Kingdom

**Keywords:** Neuroinflammation, Functional connectivity, Tau pathology, Microglia, Multimodal imaging

## Abstract

**Background:**

Neuroinflammation is implicated in Alzheimer’s disease (AD) pathogenesis, yet how changes in TSPO PET signal are associated with tau deposition, and how brain network architecture shapes this relationship remain poorly understood.

**Methods:**

We conducted a prospective, multimodal neuroimaging study in 56 participants: 35 amyloid-positive individuals across the AD spectrum (ADS) and 21 amyloid-negative healthy controls (HC). Amyloid status was classified primarily by CSF Aβ42/Aβ40 and, when eligible CSF was unavailable, by baseline amyloid PET. Participants underwent TSPO PET using [18 F]GE-180 and resting-state fMRI at baseline. A longitudinal subgroup additionally underwent follow-up tau PET using [18 F]PI-2620 (*n* = 24) and repeat TSPO PET (*n* = 18) approximately 18 months later.

**Results:**

Baseline TSPO did not differ significantly between groups. Baseline TSPO was associated with regional tau in both groups; the reciprocal association between tau and TSPO change was numerically weaker in AD, but this group difference was not statistically significant. Connectivity to baseline-TSPO epicenters was associated with higher tau in both groups, whereas connectivity to TSPO-change epicenters was associated with lower tau in AD but not controls (group-by-connectivity interaction *p* < 0.001, participant-level slope difference *p* = 0.012), a dissociation replicated in independent participants.

**Conclusion:**

These findings suggest that connectivity to established versus emerging TSPO epicenters carries distinct spatial information about tau in the AD spectrum, a pattern shaped by functional brain connectivity and absent in healthy controls. (ClinicalTrials.gov: NCT06224920)

**Supplementary Information:**

The online version contains supplementary material available at https://doi.org/10.1186/s13195-026-02230-1.

## Background

While the accumulation of misfolded amyloid-beta (Aβ) aggregates [1, 2] and intracellular hyperphosphorylated tau [3] are the pathological hallmarks of Alzheimer’s disease (AD), the central role of neuroinflammation for its development and progression is becoming increasingly recognized [4]. Recent imaging and molecular studies in AD patients have shown that microglial activation is associated with tau accumulation and Aβ deposition in vivo [5–7], and that elevated inflammatory markers in cerebrospinal fluid (CSF) and blood of AD patients are associated with tau pathology and cognitive decline [8, 9]. Experimental studies have shown that microglia can phagocytose pathological substrates including aggregated Aβ and tau [5]. In AD, distinct microglial states have been described: disease-associated microglia with a phagocytic phenotype, and neurodegenerative microglia with a dysfunctional proinflammatory phenotype [10–12]. Protective mechanisms are thought to predominate in the early stages of AD pathology [13, 14], but over time they can fail, possibly due to persistent activation and accumulation of toxic products, leading to a neurotoxic phenotype [15, 16]. Evidence suggests that in later disease stages, microglia may facilitate tau spread and Aβ plaque formation, contributing to synaptic and functional network disruption [17, 18]. However, conflicting evidence remains regarding the precise timing of these transitions and whether microglial activation is a response to Aβ and tau accumulation or a necessary driver of pathology [19, 20].

Microglial activation can be assessed in vivo using positron emission tomography (PET) tracers targeting the 18 kDa translocator protein (TSPO), a mitochondrial membrane protein primarily expressed by activated glial cells [21], thus providing an in vivo marker of a component of neuroinflammation. PET studies have reported that microglial activation is increased in AD patients [22, 23] and in patients with mild cognitive impairment [24], and further, that it persists longitudinally [25], and associates with tau accumulation in a Braak-like manner [6]. Several studies have reported that early microglial activation is associated with slower disease progression, while persistent activation is associated with more rapid decline [13, 26, 27], yet it remains unclear whether microglial activation is a secondary response to tau accumulation or an active driver of its spread, and whether this relationship varies by disease stage. Separately, tau pathology is known to spread along functionally connected brain networks [28, 29], and microglial activation itself has been shown to distribute along functional connectivity patterns [30], with TSPO-tau spatial associations extending to other tauopathies [31, 32]. Whether functional connectivity also structures the relationship between longitudinal spatial microglial dynamics and tau deposition has not been tested.

Here, we longitudinally characterized TSPO PET signal in AD-spectrum participants and healthy controls using serial TSPO PET, resting-state fMRI, and follow-up tau PET. Building on our prior observation that TSPO signal distributes along functionally connected brain regions [30], we hypothesized that functional connectivity to regions of high TSPO signal would be associated with the spatial pattern of follow-up tau burden.

## Materials and methods

### Study sample

All participants included in this study are part of the prospective cohort study “Activity of Cerebral Networks, Amyloid and Microglia in Aging and Alzheimer’s Disease” (ActiGliA), of the Munich Cluster for Systems Neuroscience at the Ludwig Maximilian University (LMU) Hospital Munich, Germany [30, 33]. Recruitment began in 2017, and was conducted in outpatient memory clinics at the Departments of Psychiatry and Psychotherapy at LMU Hospital and the Technical University of Munich, as well as the Department of Neurology and the Institute of Stroke and Dementia Research at LMU Hospital. Ethics approval was obtained from the LMU Ethics committee (Project No. 17–755), and all participants provided informed consent in accordance with the Declaration of Helsinki (ClinicalTrials.gov ID: NCT06224920, registered 2024-01-16).

The baseline cohort comprised 56 participants, including 35 AD-spectrum participants (ADS) and 21 healthy controls (HC). Amyloid status was classified primarily using the CSF Aβ42/Aβ40 ratio (Aβ positive if < 0.055; *n* = 45). When an eligible CSF ratio was unavailable (*n* = 11), amyloid status was determined by expert visual assessment of baseline amyloid PET. For [18 F]florbetaben, temporal, frontal, posterior cingulate/precuneus, and parietal cortices were assessed, and a scan was rated positive when cortical gray-matter uptake was equal to or greater than adjacent white matter and extended to the cortical margin in the majority of slices in at least one region. For [18 F]flutemetamol, frontal, posterior cingulate/precuneus, lateral temporal, inferolateral parietal, and striatal regions were assessed, and a scan was rated positive when at least one region in either hemisphere showed increased cortical signal and reduced or absent gray-white matter contrast. Eligible CSF classification took precedence; PET served only as a backup when CSF was unavailable. HC had no evidence of Aβ pathology and a baseline global Clinical Dementia Rating (CDR) score of 0. At baseline, participants underwent clinical and neuropsychological assessment, amyloid and TSPO PET, resting-state fMRI, structural MRI, and fluid biobanking. At follow-up, 24 participants (11 ADS, 13 HC) underwent tau PET and 18 (7 ADS, 11 HC) underwent repeat TSPO PET. Sixteen participants (5 ADS, 11 HC) had baseline and follow-up TSPO PET together with follow-up tau PET (Figure S1).

### Molecular and genetic phenotyping

The binding affinity of [18 F]GE-180 depends on the codominant TSPO single-nucleotide polymorphism rs6971 (Ala147Thr) [34]. Participants were genotyped as high-affinity binders (Ala/Ala), mixed-affinity binders (Ala/Thr), or low-affinity binders (Thr/Thr). Low-affinity binders (*n* = 7) and participants with missing rs6971 genotype (*n* = 3) were excluded from TSPO analyses. DNA was extracted from whole blood using the SQ Blood DNA kit (Omega Bio-Tek, USA), assessed by gel electrophoresis, amplified using a TaqMan qPCR assay, and characterized by Sanger sequencing. CSF was obtained by lumbar puncture, aliquoted, and analyzed using commercial enzyme-linked immunosorbent assays (ELISA; Fujirebio, Japan). The Aβ42/Aβ40 ratio was used for the primary amyloid classification. High- and mixed-affinity binders were retained. The primary models were not adjusted for HAB/MAB status; sensitivity models adjusted for HAB/MAB status, APOE ε4 carrier status, and both covariates together yielded materially unchanged estimates (Supplementary Table S10).

### MR imaging

All MRI scans were acquired at the Department of Radiology of the LMU Hospital using a 3T Magnetom Skyra MR system (Siemens Healthineers, Germany). A T1-weighted structural MRI sequence was obtained (0.8 mm isotropic resolution, repetition time = 2.06 s, inversion time = 1.04 s, echo time = 2.17 ms, flip angle = 12°, field of view = 24 cm), as well as a resting-state fMRI sequence with multiband acceleration factor of 6 (2.5 mm isotropic resolution, duration = 10:32, repetition time = 780 ms, echo time = 33 ms, flip angle = 50°, field of view = 21 cm).

### PET imaging

All PET scans were performed at the Department of Nuclear Medicine, LMU Hospital, using a Biograph 64 PET/CT or a harmonized Biograph mCT PET/CT scanner (Siemens Healthineers, Germany), as previously reported [30]. Participants first underwent a low-dose CT scan for attenuation correction. PET imaging was conducted using the TSPO tracer [^18^F]GE-180, the tau tracer [^18^F]PI-2620, and the Aβ tracers [^18^F]flutemetamol and [^18^F]florbetaben. Images were reconstructed using a 3-dimensional ordered subsets expectation maximization (OSEM) algorithm with a matrix size of 336 × 336 × 109 and a voxel size of 1.018 × 1.018 × 2.027 mm. Standard corrections for attenuation, scatter, decay, and random counts were applied to ensure data consistency. Amyloid PET was acquired at baseline only; no longitudinal amyloid imaging was available.

[^18^F]GE-180 (average injected dose: ~185 MBq) emission data were acquired either dynamically over 90 min or statically between 60 and 80 min post-injection to assess microglial activation, for both using the 60-80-minute post-injection window for analysis. [^18^F]PI-2620 (average injected dose: ~185 MBq) imaging was performed dynamically (0–60 min post-injection) and a 20- to 40-minute post-injection time window was extracted for tau load assessment. Amyloid-beta PET imaging was conducted using either [^18^F]flutemetamol (average injected dose: ~185 MBq) or [^18^F]florbetaben (average injected dose: ~300 MBq). Emission data were collected 90–110 min post-injection.

### PET/MRI processing

All structural MRI and PET data were processed using the Advanced Normalization Tools (ANTs) software suite (version 2.3.2) [35] and the FMRIB Software Library (FSL, version 6.0.3) [36]. T1-weighted structural MRI scans underwent bias field correction and brain extraction. For spatial normalization, the brain-extracted images were non-linearly warped to the Montreal Neurological Institute (MNI152) 2 mm isotropic template. All PET images were first rigidly registered to their respective T1-weighted MRI scans in native space. They were then spatially normalized to MNI space using the T1-to-MNI transformation parameters. For all PET images, intensity normalization was performed using the inferior cerebellar gray matter as the reference region to compute standardized uptake value ratios (SUVRs). All brain atlas-derived regions of interest (ROIs) of the gray matter masked Schaefer 200 atlas [37] were transformed from MNI152NLin6Asym to T1 space and applied to PET data for regional quantification. All co-registration and segmentation results were visually inspected for quality control. Cortical surface maps were plotted using the brainspace library in Python [38]. No partial-volume correction was applied. Gray-matter-masked ROI extraction reduces inclusion of non-gray-matter voxels but does not constitute partial-volume correction or eliminate spill-in from adjacent tissue.

### fMRI processing

Preprocessing of T1-weighted images was performed using fMRIPrep version 1.2.1 [39], built on Nipype 1.1.4 [40]. The preprocessing pipeline included intensity non-uniformity correction using the Advanced Normalization Tools (ANTs) library 2.2.0 [35], skull stripping guided by the OASIS template, and cortical surface reconstruction via FreeSurfer 6.0.1 [41]. The Schaefer 200-parcel atlas in the MNI152NLin6Asym space was used for region-based extraction of the fMRI data. The Python library nilearn [42] was applied to extract regional time-series data from the preprocessed images, while applying a band-pass filter of 0.009–0.08 Hz, detrending, a Gaussian smoothing kernel with a full-width at half-maximum of 8 mm and confound regression. Confound variables extracted from the fMRIPrep confounds file included motion parameters, white matter, cerebrospinal fluid, and their first- and second-order derivatives. The extracted time-series data were used to compute functional connectivity by estimating a Pearson correlation matrix between all brain regions defined by the atlas. The resulting correlation values were Fisher z-transformed. All further analyses were performed in Python 3.11.6, using NumPy 1.24.4 [43], pandas 2.2.3 [44], SciPy 1.11.4 [45], statsmodels 0.14.1 [46].

### Statistical analyses

The processing of MR and PET data is detailed in the Supplementary Materials. Continuous variables are reported as median values with interquartile ranges [Q1-Q3]. When reporting effects on the log scale, we additionally present geometric means and ratios (i.e., exponentiated coefficients). Statistical significance was defined as *p* < 0.05 (two-sided). Analyses used region-of-interest (ROI) data from the Schaefer 200 parcellation.

At baseline, demographics were compared between Alzheimer’s disease spectrum (ADS) participants and healthy controls (HC) using Mann-Whitney U tests for continuous variables and Fisher’s exact tests for categorical variables. To compare PET data at the group level, we computed standardized SUVRs by z-scoring the ADS baseline and follow-up TSPO PET SUVRs, and HC follow-up TSPO SUVRs, using the HC TSPO baseline ROI SUVR distribution. Follow-up tau SUVRs for ADS were z-scored by ROI using the follow-up HC ROI distribution. For descriptive group-level comparisons and cortical maps, follow-up-minus-baseline ROI z-score differences were computed per participant; these are referred to as standardized TSPO z-score differences rather than TSPO change. These z-scored values were used only for the descriptive group-level comparisons and cortical visualizations, while mixed effects analyses used log-transformed SUVRs without z-scoring to the HC distribution. Analyses were classed as primary hypothesis driven, exploratory, or sensitivity. We applied Benjamini-Hochberg correction across the primary hypothesis-driven tests (baseline TSPO and TSPO change with tau, the reciprocal association, the connectivity-tau associations, and their group interactions), which are reported with exact p-values and confidence intervals, with q-values reported in the Supplement. Exploratory analyses (network- and Braak-stratified moderation) were corrected within each family, reporting corrected and uncorrected p-values (Tables S4–S7). Sensitivity analyses (epicenter thresholds re-estimation and leave-one-subject-out) re-test existing hypotheses and were not corrected.

To investigate differences in microglial activation between groups, we tested the association of TSPO at follow-up between ADS and HC participants using linear regression (reporting standardized β with p-values) and Spearman’s rank correlation (ρ). Similarly, we tested the association between the descriptive standardized TSPO z-score difference within ADS and the baseline TSPO levels. To assess the relationship between microglial activation and tau levels in ADS, we tested the association between baseline TSPO and follow-up tau levels, including their pairwise covariance across ROIs. Finally, we correlated the baseline TSPO levels and TSPO change levels against the functional connectivity to the tau epicenter and the TSPO change epicenters. For the primary analyses, epicenters were defined as the top 20% of that participant’s ROIs by the relevant tracer SUVR, while for the out-of-sample validation, epicenters were defined as the top 20% of ROIs ranked by group-mean baseline TSPO SUVR (or by group-mean TSPO change for change epicenters). For each participant and each target ROI, we computed the mean resting-state functional connectivity between that target ROI and all epicenter ROIs (i.e., connectivity from each region to the epicenter set across the whole brain, excluding self-connections). This yielded one FC-to-epicenter value per target ROI per participant.

Subsequently, subject-level associations between tau and TSPO measures were evaluated with linear mixed-effects (LME) models estimated by restricted maximum likelihood (REML). Unless stated otherwise, models included the relevant fixed effects, and crossed random intercepts for subject and ROI, to account for both region-to-region and participant-to-participant variation in TSPO uptake, which prevents treating the ROIs within a participant as independent observations. PET SUVRs were log-transformed for LME analyses. Longitudinal change in TSPO was summarized as the log of the follow-up/baseline SUVR ratio (hereafter TSPO change). We first compared baseline TSPO between ADS and HC, and then compared TSPO change between groups, with the crossed mixed effects model, as well as the mean SUVRs with a Welch’s t-test. To examine spatial patterning and moderation, we included factor terms for Braak stage (i.e., the Braak stage that a ROI is implicated in, stages I, III-VI) and the Yeo-7 functional network parcellation in models fitted separately within ADS and HC; reference levels were Braak I and the Control network. Interaction terms tested whether the TSPO-tau slope varied by Braak stage and by network. We also modeled TSPO change as a function of baseline TSPO (and group), and modeled follow-up tau as a function of baseline TSPO and TSPO change (including their interaction).

To test whether functional network architecture modulates PET tracer patterns, we assessed how regional PET SUVRs covaried with functional connectivity (FC). Epicenter ROIs were defined as the top 20% of regions by follow-up tau SUVR, baseline TSPO SUVR, or TSPO change. For each epicenter set, we computed FC to the remaining ROIs and tested within-tracer and cross-tracer associations, estimating group-specific effects in ADS and HC. Cross-modal models were fitted first with each connectivity predictor alone and then with both predictors entered simultaneously. For the key between-group comparison, we additionally fitted a model with participant-specific random slopes for connectivity and compared the individual regional slopes between groups using Welch’s t-test. Sensitivity analyses re-estimated the cross-modal LME at epicenter thresholds of 5%, 10%, 15%, and 20%. Robustness was assessed using leave-one-subject-out analyses and covariate adjustment for APOE ε4 carrier status, HAB/MAB binding status, and both together (Supplementary Table S10).

### Out-of-sample validation

To assess whether the connectivity-tau associations generalized beyond the longitudinal subgroup, we used partially non-overlapping imaging samples (Figure S1). Baseline-TSPO epicenters (top 20% of ROIs by group-mean SUVR) were defined using 15 ADS participants with baseline TSPO PET and fMRI who did not undergo tau PET. TSPO-change epicenters (top 20% by group-mean follow-up/baseline TSPO ratio) were defined using 6 ADS participants with baseline and follow-up TSPO PET. The primary validation analysis used baseline fMRI in the 10 ADS participants and 13 HC with follow-up tau PET and usable baseline fMRI; five of these ADS participants contributed to neither epicenter definition and therefore constituted a fully independent validation sample. The eleventh ADS participant with follow-up tau PET lacked baseline fMRI but had follow-up fMRI. We therefore repeated the analysis as a sensitivity analysis using follow-up fMRI in all 11 ADS participants; six ADS participants were then independent of both epicenter definitions (Supplementary Tables S3 and S11). We additionally tested whether the cross-sectional ADS-HC difference in baseline TSPO predicted longitudinal TSPO change and whether connectivity to regions with the largest ADS-HC deficit (pseudo-change epicenters, bottom 20%) was associated with follow-up tau.

## Results

We studied 56 participants (35 amyloid-positive AD-spectrum participants [ADS], 21 amyloid-negative healthy controls [HC]). Amyloid status was established using CSF Aβ42/Aβ40 in 45 participants and baseline amyloid PET as a backup in 11 participants with unavailable eligible CSF. A subgroup underwent follow-up tau PET (*n* = 24; 11 ADS, 13 HC) and repeat TSPO PET (*n* = 18; 7 ADS, 11 HC) approximately 18 months later, yielding 16 participants with complete longitudinal TSPO and follow-up tau data (Table 1 and Figure S1). Participants were well matched for age, sex, weight, and years of education. As expected, ADS participants had poorer cognitive performance and higher CSF total tau and p-tau181 together with lower CSF Aβ42/Aβ40. Demographic, clinical, and biomarker characteristics of the complete longitudinal subgroup are reported in Supplementary Table S1.

**Table 1:** Demographics and clinical data at baseline

Characteristic	ADS	*n*	HC	*n*	*p*
Age (years)	72.0 [69.0, 77.5]	35	69.0 [67.0, 72.0]	21	0.136
Sex, female	20 (57.1%)	35	12 (57.1%)	21	1.000
Weight (kg)	69.0 [60.0, 76.0]	33	78.0 [65.0, 88.0]	21	0.065
Education (years)	15.0 [11.5, 17.0]	35	13.0 [12.0, 17.0]	21	0.792
MMSE	26.0 [23.0, 28.5]	35	30.0 [29.0, 30.0]	21	< 0.001
CDR-SOB	2.2 [0.5, 3.0]	34	0.0 [0.0, 0.0]	21	< 0.001
Global CDR	0.5 [0.0, 0.5]	34	0.0 [0.0, 0.0]	21	< 0.001
CERAD-NB	65.8 [51.9, 78.5]	34	86.0 [82.0, 89.5]	21	< 0.001
CSF Aβ42 (pg/mL)	453.4 [333.5, 583.9]	26	1009.0 [590.2, 1422.5]	19	< 0.001
CSF Aβ42/40 ratio	0.03 [0.03, 0.04]	26	0.08 [0.07, 0.08]	19	< 0.001
CSF p-tau181 (pg/mL)	86.4 [75.0, 102.7]	27	50.2 [40.7, 61.0]	19	< 0.001
CSF t-tau (pg/mL)	465.3 [398.0, 629.7]	28	222.0 [177.4, 321.4]	19	< 0.001
APOE ε4 carriers	24 (75.0%)	32	5 (23.8%)	21	< 0.001
rs6971 LAB status	6 (18.8%)	32	1 (4.8%)	21	0.223

### TSPO binding is comparable between ADS and HC at baseline

**Fig. 1:**
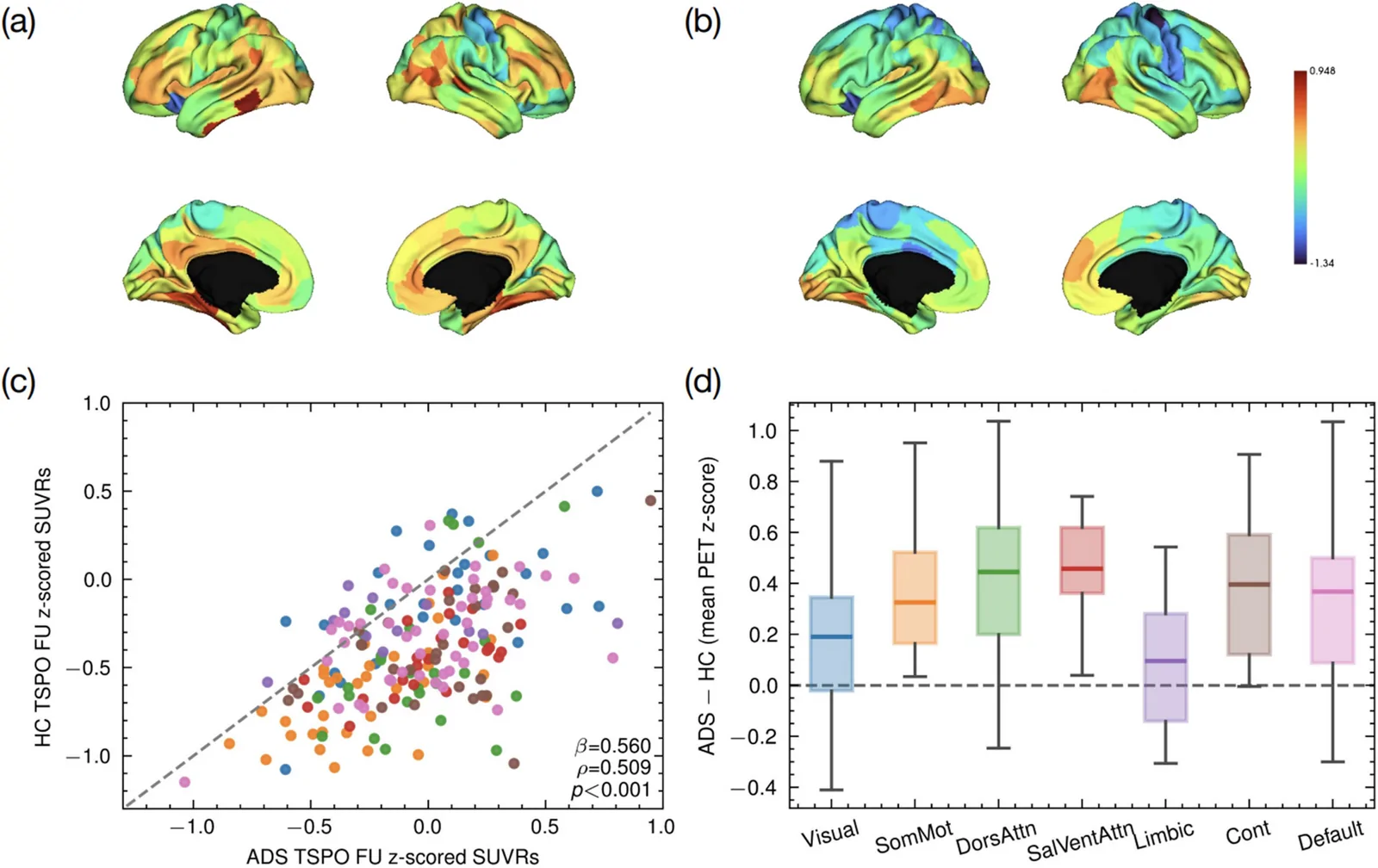
Microglial activation at follow-up varies between ADS and HC. Legend: **a** ADS and **b** HC cortical surface maps of mean TSPO SUVRs at follow-up z-scored against the HC baseline levels. **c** ROI-wise plot of mean TSPO z-scored SUVRs at follow-up for ADS against HC levels, with **d** differences between ADS-HC visualized by functional network

At baseline, ADS participants had similar TSPO binding to age-matched HC. At the participant level, mean SUVR was 0.96 in ADS and 0.98 in HC (Welch t = − 0.89, *p* = 0.38, *n* = 25 ADS, 20 HC). This was also evident in mixed-effects models of log(TSPO) at baseline (HC/ADS ratio = 1.022, 95% CI 0.98–1.07, *p* = 0.355). In ADS, the limbic (exp(β) = 1.19, 95% CI 1.10–1.28) and visual networks (1.24, 95% CI 1.17–1.31) showed the highest TSPO relative to the control network reference. HC showed a similar pattern (limbic: 1.22, 95% CI 1.15–1.28, visual: 1.22, 95% CI 1.15–1.28). No individual network differed significantly between groups (all q > 0.5, see Fig. 1).

### Longitudinal TSPO change is comparable between groups

**Fig. 2:**
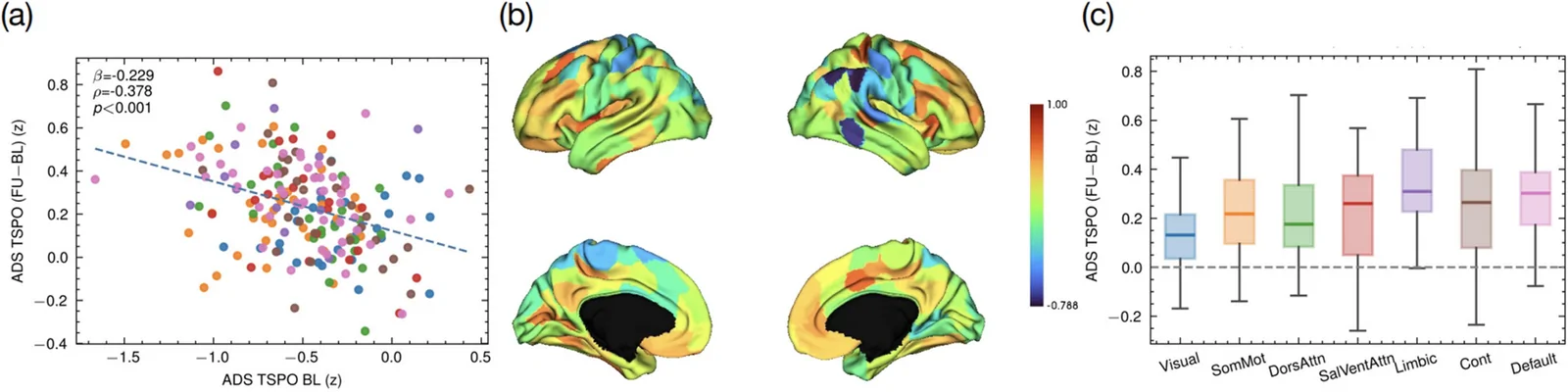
Association of mean ADS TSPO SUVR at baseline with the TSPO change from follow-up to baseline. Legend: **a** Baseline TSPO ADS z-scored SUVR values are negatively associated with the ROI-wise mean difference between follow-up and baseline TSPO SUVRs. **b** Cortical surface plot of the TSPO SUVR difference from follow-up to baseline (all ROI values z-scored to TSPO HC baseline) and **c** summarized by functional networks

Over 18 months, TSPO change increased modestly in ADS (mean 0.041) and decreased in HC (mean − 0.06), but without a statistically significant difference between groups (Welch t = 1.18, *p* = 0.26), similarly when adjusting for baseline TSPO (*p* = 0.53). Within the ADS group, median differences between follow-up and baseline SUVR z-scores were positive across functional networks, with the highest increase in the limbic network (median z difference = 0.31, IQR 0.23–0.48, Fig. 2). Higher baseline TSPO was associated with less subsequent TSPO change in both groups (exp(β) = 0.89, *p* < 0.001, ρ = −0.38 in ADS, exp(β) = 0.78 *p* < 0.01 in HC).

This negative baseline-change relationship varied by network: in FDR-corrected analyses, the plateau was significantly attenuated in the visual network in ADS, and in the visual and dorsal-attention networks in HC (all q < 0.05, Table S4). By Braak stage, both groups showed the steepest negative slopes at Braak I, weakening progressively at higher stages (all q < 0.05, Table S5).

### Baseline TSPO is associated with regional tau in ADS and HC

We then examined whether baseline TSPO signal was associated with tau burden measured at follow-up. Because tau PET signal is low in amyloid-negative individuals, we first verified that regional tau in HC reflected biological variability rather than noise before testing tau associations. In HC, tau SUVR in Braak I, III and IV regions significantly exceeded the cerebellar reference (49 ROIs, geometric mean 1.15 (1.14) *p* < 0.001), and tau SUVR was higher in Braak I–IV than Braak V–VI regions in every HC participant (13/13; geometric mean 1.15 vs. 1.06; paired t = 9.4, *p* < 0.001).

**Fig. 3:**
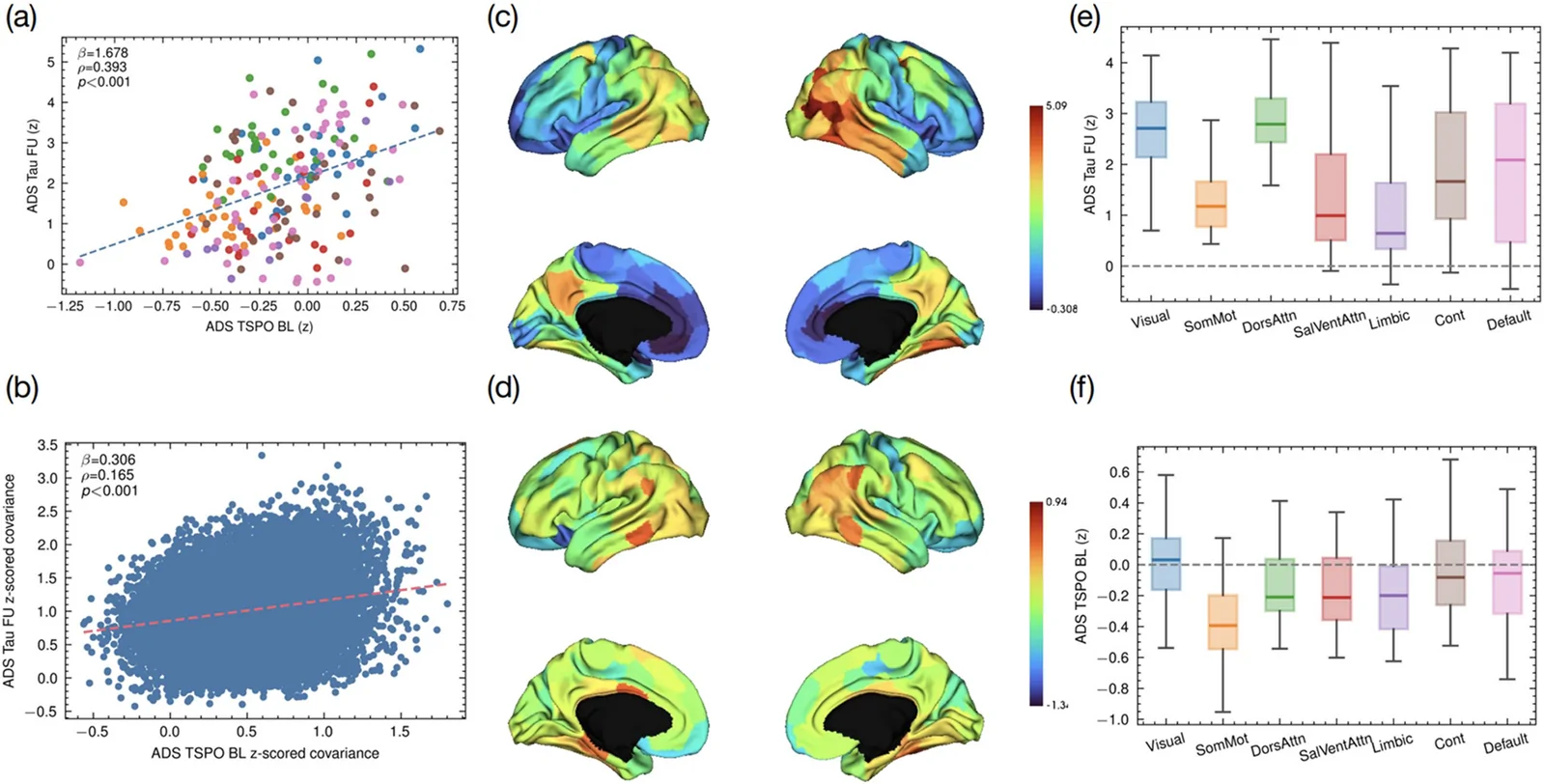
Association between microglial activation and tau levels in ADS participants. Legend: **a** ROI-wise z-scored ADS tau SUVRs at follow-up are positively associated with z-scored TSPO SUVRs at baseline. **b** ADS tau ROI pairwise covariance is also positively associated with TSPO SUVRs at baseline covariance. **c** Surface cortical plot of ADS tau at follow-up z-scored ROI-wise against HC tau, and of **d** ADS TSPO at baseline z-scored against HC at baseline, **e** Summary of mean z-scored tau SUVRs at follow-up by functional network and **f** TSPO at baseline against HC

At the group level, brain regions with higher baseline TSPO showed higher tau at follow-up in ADS (β = 1.68, *p* < 0.001, ρ = 0.39), and the spatial patterns of TSPO and tau covaried positively across ROIs (β = 0.31, *p* < 0.001, ρ = 0.17, Fig. 3). At the subject level, both higher baseline TSPO (exp(β) = 1.91, 95% CI 1.77–2.07) and higher TSPO change (1.82, 95% CI 1.56–2.14) were associated with higher tau in ADS (both *p* < 0.001). Their interaction was negative but not significant (exp(β) = 0.86, 95% CI 0.46–1.60, *p* = 0.64). In HC the corresponding interaction was significant (exp(β) = 0.86, 95% CI 0.83–0.90, Table 2A).

**Table 2:** Follow-up tau as a function of baseline TSPO and TSPO change

A) log(tau) ~ log(TSPO baseline) × TSPO change						
**Variable**	ADS			HC		
	**β [95% CI]**	**exp(β) [95% CI]**	**p**	**β [95% CI]**	**exp(β) [95% CI]**	**p**
TSPO baseline	0.647 [0.57, 0.73]	1.91 [1.77, 2.07]	< 0.001	0.412 [0.38, 0.44]	1.51 [1.47, 1.55]	< 0.001
TSPO change	0.601 [0.44, 0.76]	1.82 [1.56, 2.14]	< 0.001	0.131 [0.10, 0.16]	1.14 [1.11, 1.18]	< 0.001
TSPO BL × change	−0.150 [− 0.77, 0.47]	0.86 [0.46, 1.60]	0.637	−0.145 [− 0.19, − 0.10]	0.86 [0.83, 0.90]	< 0.001

B) TSPO change ~ log(TSPO baseline) + log(tau)						
**Variable**	**ADS**			**HC**		
	**β [95% CI]**	**exp(β) [95% CI]**	**p**	**β [95% CI]**	**exp(β) [95% CI]**	**p**
TSPO baseline	−0.159 [− 0.19, − 0.13]	0.85 [0.83, 0.88]	< 0.001	−0.335 [− 0.38, − 0.29]	0.72 [0.68, 0.75]	< 0.001
tau follow-up	0.079 [0.06, 0.10]	1.08 [1.06, 1.11]	< 0.001	0.226 [0.17, 0.28]	1.25 [1.19, 1.32]	< 0.001

In the reciprocal model, higher regional tau was associated with greater TSPO change in ADS (exp(β) = 1.08, 95%CI 1.06–1.11, *p* < 0.001) and in HC (exp(β) = 1.25, 95%CI 1.19–1.32, *p* < 0.001). The association was numerically steeper in HC than in ADS, but this group difference was not supported by participant level comparison of individual slopes (*p* = 0.15). This TSPO-tau association was network-dependent: in ADS, the TSPO-tau slope was non-significant in the reference (control) network (*p* = 0.37), and no network showed a statistically significant contribution (Table S6), whereas in HC the association was significant only for the visual regions (Table S6). By Braak stage, the TSPO-tau slope was strongest at Braak IV in ADS, with no stage significant in HC (Tables S6-S7).

### Functional connectivity to TSPO epicenters is associated with the spatial pattern of tau

**Fig. 4:**
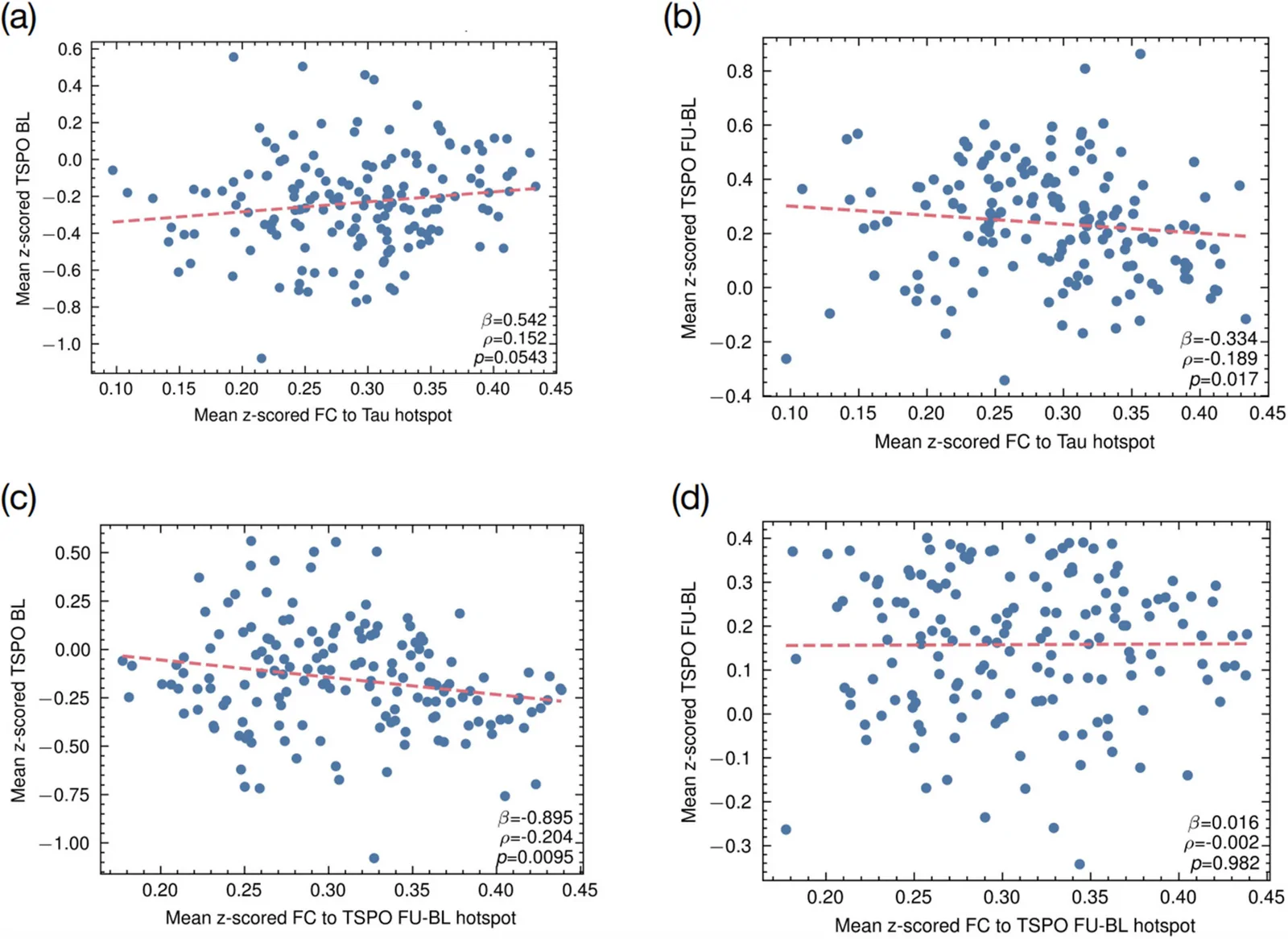
Association of mean ADS TSPO SUVR and the difference between follow-up and baseline TSPO SUVRs with FC to tau and TSPO change epicenters. Legend: Correlation between (**a**) TSPO at baseline or (**b**) TSPO change and connectivity to tau epicenter. (**c**) Negative association between TSPO and FC to TSPO change epicenter. (**d**) Correlation between TSPO change and FC to the TSPO change epicenter.

We next examined whether functional connectivity modulated the spatial relationship between TSPO binding and tau. At the group level, exploratory analyses showed that baseline TSPO showed no significant association with connectivity to tau epicenters (ρ = 0.15, *p* = 0.054, Fig. 4a), while TSPO change was negatively associated with connectivity to tau epicenters (β = −0.33, *p* = 0.017, Fig. 4b). Baseline TSPO was negatively associated with connectivity to TSPO change epicenters (β = −0.90, *p* = 0.0095, Fig. 4c), while TSPO change showed no association with connectivity to its own epicenters at the group level (β = 0.02, *p* = 0.98, Fig. 4d). Proximity to tau epicenters and proximity to TSPO change epicenters were inversely related in ADS (ρ = −0.27, *p* < 0.001) but not HC (*p* = 0.054), suggesting that the two microglial landscapes occupy distinct connectivity neighborhoods.

At the subject level, we first confirmed that each PET signal tracked connectivity to its own epicenters: tau followed tau epicenter connectivity in both groups (ADS exp(β) = 2.68, HC 1.66, both *p* < 0.001), and TSPO change followed its own epicenter connectivity in ADS (1.12, *p* < 0.001) but not HC (*p* = 0.127). We then tested whether connectivity to TSPO epicenters was associated with the spatial distribution of tau. In single-predictor models, connectivity to baseline TSPO epicenters was associated with higher tau in both groups (ADS exp(β) = 1.51, 95% CI 1.33–1.71, HC 1.06, 95% CI 1.01–1.10, both *p* < 0.001), while connectivity to TSPO change epicenters was associated with lower tau in ADS (0.72, 95% CI 0.63–0.82, *p* < 0.001) but not in HC (1.00, 95% CI 0.95–1.04, *p* = 0.86, Table 3A). This group difference in the TSPO change epicenter association was significant (group-by-connectivity interaction exp(β) = 1.34, 95% CI 1.21–1.48, *p* < 0.001) and was corroborated by a participant-level comparison of individual slopes (mean difference − 0.53, Welch t = − 3.10, *p* = 0.012, all five patients showing a negative slope). When both predictors were entered together, the change-epicenter association in ADS was attenuated but remained significant (0.83, 95% CI 0.72–0.97, *p* = 0.020, Table 3B), and remained significant in four of five leave-one-subject-out analyses, although the non-significant fold underscores the uncertainty in this small subgroup, while the baseline TSPO effect persisted in both groups (ADS 1.23, 95% CI 1.05–1.44, HC 1.07, 95% CI 1.02–1.12, both *p* < 0.001, Table 3B). This cross-modal dissociation was robust to the choice of epicenter threshold, with the TSPO change connectivity effect remaining negative and significant in ADS across all thresholds tested (5–20%, Table S2).

**Table 3:** TSPO change, tau, and connectivity-dependent associations

A) Single-predictor cross-modal models: log(tau) ~ FC						
**Variable**	ADS			HC		
	**β [95% CI]**	**exp(β) [95% CI]**	**p**	**β [95% CI]**	**exp(β) [95% CI]**	**p**
FC to TSPO BL epicenters	0.410 [0.29, 0.53]	1.51 [1.33, 1.71]	< 0.001	0.054 [0.013, 0.10]	1.06 [1.01, 1.10]	< 0.001
FC to TSPO change epicenters	−0.328 [− 0.45, − 0.20]	0.72 [0.63, 0.82]	< 0.001	-0.004 [− 0.05, 0.04]	1.00 [0.95, 1.04]	0.857

B) Multivariate models: log(tau) ~ FC to TSPO change epicenters + FC to TSPO BL epicenter						
**Variable**	**ADS**			**HC**		
	**β [95% CI]**	**exp(β) [95% CI]**	**p**	**β [95% CI]**	**exp(β) [95% CI]**	**p**
FC to TSPO BL epicenter	0.205 [0.05, 0.36]	1.23 [1.05, 1.44]	0.012	0.069 [0.02, 0.12]	1.07 [1.02, 1.12]	0.005
FC to TSPO change epicenter	−0.182 [− 0.33, − 0.03]	0.83 [0.72, 0.97]	0.020	0.007 [− 0.05, 0.06]	1.01 [0.96, 1.06]	0.799

### Group-level TSPO connectivity patterns are associated with tau out of sample

To test generalization beyond the complete longitudinal subgroup, baseline-TSPO epicenters were defined from 15 ADS participants with baseline TSPO PET and fMRI who did not contribute tau PET data, while TSPO-change epicenters were defined from 6 ADS participants with repeat TSPO PET (Figure S2; Supplementary Tables S8-S9). In the primary validation using baseline fMRI, connectivity to baseline-TSPO epicenters was associated with higher regional tau in ADS (exp(β) = 2.34, 95% CI 1.97–2.78, *p* < 0.001, *n* = 10) and in HC (exp(β) = 1.48, 95% CI 1.41–1.55, *p* < 0.001, *n* = 13; Supplementary Table S3). Among the five ADS participants independent of both epicenter definitions, single-predictor models showed higher tau with greater FC to baseline-TSPO epicenters (exp(β) = 2.90, 95% CI 2.26–3.73) and lower tau with greater FC to TSPO-change epicenters (exp(β) = 0.38, 95% CI 0.34–0.43; both *p* < 0.001); both effects remained significant in the combined model. Because one additional ADS participant had follow-up but not baseline fMRI, we repeated the validation using follow-up fMRI for all 11 ADS participants. The single-predictor associations remained significant, including in the six fully independent ADS participants. In the combined models, however, the TSPO-change-epicenter term was attenuated and no longer significant (independent ADS: exp(β) = 0.90, 95% CI 0.73–1.10, *p* = 0.299; all ADS: exp(β) = 0.93, 95% CI 0.79–1.09, *p* = 0.345; Supplementary Table S11).

We then asked whether the spatial information carried by TSPO change epicenters could be approximated from baseline imaging. Across the 200 ROIs, the cross-sectional difference in baseline TSPO between ADS and HC (computed from all 26 ADS and 20 HC with baseline MRI and TSPO PET) was negatively associated with actual longitudinal TSPO change in the subgroup with repeat scans (ρ = −0.19, *p* = 0.007): regions where ADS participants had the lowest TSPO relative to HC at baseline subsequently showed the greatest TSPO increase. We therefore defined pseudo-change epicenters as the 20% of ROIs where the ADS-HC baseline deficit was largest and tested whether connectivity to these regions carried the same tau-predictive information as connectivity to the actual TSPO change epicenters. Each ADS tau participant’s connectivity to these pseudo-change epicenters was associated with lower follow-up tau at the group level (ρ = −0.57, *p* < 0.001), closely mirroring the pattern obtained with the actual longitudinal TSPO change epicenters (ρ = −0.64). This is a spatial association and does not reflect a statistically significant group difference in overall TSPO.

## Discussion

Neuroinflammation is a well-established feature of both AD and aging, and TSPO PET provides an in-vivo marker of a component of this process. In this study, we longitudinally characterized TSPO PET signal and examined its relationship to tau levels and functional connectivity. We report four principal findings. First, baseline TSPO signal did not differ significantly between groups, and longitudinal TSPO change did not differ significantly over 18 months between groups. Second, baseline TSPO was spatially associated with follow-up tau in both groups. Third, functional connectivity revealed that baseline TSPO and TSPO change carried independent spatial information about tau in ADS: connectivity to the former was associated with higher tau, while connectivity to the latter was associated with lower tau, a dissociation absent in HC. Fourth, the critical TSPO-change association was confirmed in participants who contributed to neither epicenter definition. Together, these results suggest that established and emerging TSPO signal represent spatially distinct processes in AD-spectrum participants, compatible with a difference between predominantly local TSPO-tau coupling in healthy aging and a more spatially reorganized inflammatory pattern in the AD spectrum.

### Microglial activation trajectories in ADS versus healthy aging

At baseline and over follow-up, mean TSPO did not differ significantly between groups, and we therefore do not interpret a group-level difference in level or trajectory. The lack of a between-group difference should be interpreted with caution: differences in cerebral blood flow, together with the blood-brain barrier penetration characteristics of [¹⁸F]GE-180, may contribute to the observed signal, as discussed below. We therefore base our primary inferences on within-group spatial associations. Related studies have proposed models of stage-dependent microglial activation, in which initially protective or compensatory responses may give way to more dysfunctional inflammatory states during AD progression [26, 47, 48]. The exact reasons for this remain unclear, but one possibility is that chronic exposure to AD pathology promotes a dysfunctional inflammatory state with reduced capacity to clear aggregated proteins [49, 50]. Alternatively, the overall increase may mask regional heterogeneity: some regions may harbor chronically activated, dysfunctional microglia unable to clear aggregated proteins [12, 51], while others may fail to mount an adequate inflammatory response to rising tau, resulting in continued but insufficient inflammation. Furthermore, increased baseline TSPO was associated with reduced subsequent TSPO increase in both groups, with some network-specific changes, which is compatible with a plateau-like pattern in TSPO signal, though with network-specific variation: in ADS, this plateau was attenuated in the visual network, compatible with continued TSPO signal in regions with the highest pathological burden. This inverse baseline-to-change relationship could partly reflect regression to the mean, particularly at the level of global TSPO signal, as change was expressed relative to baseline.

### Microglia-tau coupling

In both groups, higher baseline TSPO was associated with higher follow-up tau, and TSPO change was positively associated with tau. However, the reciprocal tau–TSPO-change association was numerically stronger in HC, though this group difference was not statistically significant at the participant level [6, 52]. Any such difference, if confirmed, would be compatible with reduced tau-associated inflammatory upregulation, though it was not statistically significant here and causal directionality cannot be inferred. The TSPO-tau association in HC is unlikely to reflect only floor-level signal because regional tau exceeded the cerebellar reference in early-to-intermediate Braak regions; however, in amyloid-negative older adults this pattern is compatible with primary age-related tauopathy (PART) and should not be interpreted as validation of an AD-specific cascade. Chronic activation may drive microglia toward an exhausted phenotype with diminished phagocytic capacity [12, 49], while tau itself can induce microglial senescence [53, 54], potentially creating a cycle in which the inflammatory response becomes increasingly disconnected from actual tau pathology.

### Connectivity-dependent interactions between tau and microglia

Given evidence that pathological tau spreads along connected neural networks [29] and that microglial activation can also follow brain connectivity patterns [30], we examined whether functional connectivity was associated with the spatial relationship between longitudinal TSPO signal and tau deposition. Consistent with this framework, we observed that brain regions strongly connected to baseline TSPO “epicenters” contained higher tau at follow-up in both ADS and HC participants. By contrast, connectivity to TSPO change epicenters showed a divergent association. In ADS, greater connectivity to TSPO change epicenters was associated with lower tau even after accounting for connectivity to baseline TSPO epicenters, whereas no such association was observed in HC. One interpretation is that baseline TSPO epicenters mark regions embedded in tau-vulnerable networks, whereas TSPO-change epicenters identify regions where inflammatory activity is located outside the dominant tau-associated network architecture.

These findings are compatible with a shift from predominantly local TSPO-tau coupling in healthy aging toward a more spatially reorganized inflammatory pattern in ADS participants. In HC, the relationship between tau and TSPO change appeared predominantly local, as tau-rich regions show stronger TSPO increases, while in ADS, longitudinal increases in TSPO are largely spatially distinct from connectivity-associated regional tau, compatible with different or competing contributors to regional TSPO signal. Experimental studies support this interpretation: microglia can internalize and degrade both tau and Aβ [55, 56], and activated microglia or T cells can dampen tau-mediated neurodegeneration [5]. However, when microglia become dysfunctional, they can facilitate rather than restrain tau spread [57, 58], and tau itself induces microglial senescence [53, 54], potentially explaining the spatial decoupling we observe in ADS. The absence of this pattern in HC is compatible with an ADS-related difference, but the small subgroups and observational design preclude attributing it specifically to disease-related disruption. Importantly, these connectivity-associated patterns replicated in independent participants; baseline-TSPO epicenters were defined entirely independently of the tau sample, and the critical TSPO-change association was confirmed in participants who contributed to neither epicenter definition. Furthermore, network- and Braak-stratified effects largely did not survive correction, consistent with our early-disease sample.

### Strengths and limitations

The strengths of this study include our longitudinal study design, allowing us to capture changes in neuroinflammatory TSPO signal, alongside cross-sectional imaging data for tau. This is one of the largest cohorts that has undergone TSPO-PET imaging alongside tau PET. Combined with multi-modal neuroimaging, we were able to characterize associations between longitudinal TSPO signal, tau levels and functional connectivity. Using patient-specific fMRI-derived functional connectivity measures is a key advantage of our approach, as tau and microglial distributions in ADS may show considerable variability, with recent work identifying several distinct ‘trajectories’ tau spread can take [59]. Finally, careful biomarker-based stratification was performed, to compensate for the lack of pathological verification of clinical diagnoses. We further leveraged the partially non-overlapping availability of imaging modalities to conduct out-of-sample validation, confirming that the within-sample connectivity-tau associations replicated in participants whose data were not used to define TSPO epicenters. Additionally, the spatial pattern of the (non-significant) baseline ADS-HC difference in TSPO was associated with subsequent TSPO change, and connectivity to the corresponding pseudo-change epicenters carried tau-related information similar to the actual longitudinal epicenters. Limitations include the sample size after strict inclusion criteria and complete-data requirements, but this had to be balanced against the risk of having unclear diagnostic groups. While we acquired longitudinal imaging data for TSPO PET, tau PET was only available at follow-up, and therefore no longitudinal tau analysis was possible. Longitudinal dynamics across the entire progression of the ADS are also difficult to infer, as our dataset emphasizes the early stages of the disease. Our ADS group spanned the early AD continuum, from preclinical (Aβ-positive, CDR 0) to mild cognitive impairment and mild dementia; a preclinical individual may be considerably earlier in the disease than a MCI patient, and these stages may differ in their neuroinflammatory profiles, but the sample size did not permit stable stage-stratified analyses. Another limitation relates to the TSPO-PET tracer [18 F]GE-180, which has been reported to exhibit relatively limited blood-brain barrier penetration compared with other second-generation TSPO tracers. This characteristic may influence the interpretation of absolute TSPO signal levels and warrants caution when comparing absolute SUVR values across groups. However, the primary findings of the present study are based on within-group spatial and longitudinal associations rather than absolute group differences. Importantly, [18 F]GE-180 has demonstrated concordance with immunohistochemical markers of microglial activation in Alzheimer’s disease models, and its regional distribution has been shown to reflect established patterns of neuroinflammation in human neurodegenerative disorders. Furthermore, low-affinity TSPO binders were excluded from all analyses. Nevertheless, replication using alternative microglial PET tracers with more favorable kinetic properties will be important to further validate the present findings. Furthermore, although TSPO reflects an established biomarker of neuroinflammation, more specific targets for distinct microglia phenotypes, such as TREM2 [60] or P2RY12 [61] need to be investigated to characterize regional neuroinflammation in AD. The central longitudinal analyses were based on only five ADS and 11 HC participants with complete repeat TSPO and follow-up tau data. Region-level mixed-effects models account for clustering across participants and ROIs but do not increase the number of independent participants; consequently, the connectivity-epicenter and network/Braak-stratified findings should be considered exploratory and hypothesis-generating, with limited generalizability. The complete-data ADS subgroup comprised four participants with CDR 0.5 and one with CDR 1 and did not include a preclinical participant, further limiting inference across the AD continuum. No partial-volume correction was applied, and gray-matter masking is not a substitute for correction; atrophy and spill-in may therefore have influenced cortical SUVRs. In addition, inferior cerebellar gray matter was used as the SUVR reference without arterial input. Although relatively spared in early AD, this region cannot be assumed to be completely unaffected across the disease spectrum, and alternative normalization strategies should be assessed in future work. Restriction to cortical Schaefer ROIs also does not exclude tracer off-target binding or spill-in from adjacent structures. Residual confounding by sex-related factors, hormone-replacement therapy, cerebrovascular disease and vascular risk, systemic inflammation, and medications cannot be excluded.

In conclusion, in our sample, established and emerging TSPO signal in Alzheimer’s disease occupies a spatially distinct network neighborhood and carries distinct information about the regional tau distribution. In this exploratory sample, this pattern differed from that in healthy controls and was reproduced in participants not used to define the relevant epicenters, suggesting that functional connectivity is associated with a spatial pattern in which established and emerging TSPO signal relate differently to tau. These findings highlight the potential of connectivity-informed neuroinflammatory biomarkers for characterizing the spatial distribution of tau and provide a framework for future studies testing whether connectivity-informed neuroinflammatory patterns have prognostic or treatment-response relevance. Future studies should test whether this dissociation intensifies across later disease stages, whether it is replicated with more specific microglial tracers, and whether the connectivity-defined inflammatory landscapes identified here predict differential responses to immunomodulatory interventions.

## Supplementary Information

Supplementary Material 1.

## Data Availability

Data will be made available upon reasonable request and in compliance with data protection regulations. Analysis code will be made available on GitHub upon publication.

## References

[1] Hardy J, Allsop D. Amyloid deposition as the central event in the aetiology of Alzheimer’s disease. Trends Pharmacol Sci. 1991;12:383–8. 10.1016/0165-6147(91)90609-v. 1763432

[2] Hardy JA, Higgins GA. Alzheimer’s disease: the amyloid cascade hypothesis. Science. 1992;256:184–5. 10.1126/science.1566067. 1566067

[3] Braak H, Braak E. Neuropathological staging of Alzheimer-related changes. Acta Neuropathol (Berl). 1991;82:239–59. 10.1007/BF00308809. 1759558

[4] Leng F, Edison P. Neuroinflammation and microglial activation in Alzheimer disease: where do we go from here? Nat Rev Neurol. 2021;17:157–72. 10.1038/s41582-020-00435-y. 33318676

[5] Gratuze M, Chen Y, Parhizkar S, Jain N, Strickland MR, Serrano JR, et al. Activated microglia mitigate Aβ-associated tau seeding and spreading. J Exp Med. 2021;218:e20210542. 10.1084/jem.20210542. PMC8190588 34100905

[6] Pascoal TA, Benedet AL, Ashton NJ, Kang MS, Therriault J, Chamoun M, et al. Microglial activation and tau propagate jointly across Braak stages. Nat Med. 2021;27:1592–9. 10.1038/s41591-021-01456-w. 34446931

[7] Malpetti M, Kievit RA, Passamonti L, Jones PS, Tsvetanov KA, Rittman T, et al. Microglial activation and tau burden predict cognitive decline in Alzheimer’s disease. Brain J Neurol. 2020;143:1588–602. 10.1093/brain/awaa088. PMC7241955 32380523

[8] Rauchmann B-S, Sadlon A, Perneczky R, Alzheimer’s Disease Neuroimaging Initiative. Soluble TREM2 and Inflammatory Proteins in Alzheimer’s Disease Cerebrospinal Fluid. J Alzheimers Dis JAD. 2020;73:1615–26. 10.3233/JAD-191120. 31958095

[9] Campanelli L, Galeano P, Prestia FA, Cuesta C, Dalmasso MC, Flores-López M et al. Blood levels of cytokines highlight the role of inflammation in Alzheimer’s disease. Heliyon. 2025;11. 10.1016/j.heliyon.2025.e41725. PMC11770505 39872450

[10] Friedman BA, Srinivasan K, Ayalon G, Meilandt WJ, Lin H, Huntley MA, et al. Diverse Brain Myeloid Expression Profiles Reveal Distinct Microglial Activation States and Aspects of Alzheimer’s Disease Not Evident in Mouse Models. Cell Rep. 2018;22:832–47. 10.1016/j.celrep.2017.12.066. 29346778

[11] Gerrits E, Brouwer N, Kooistra SM, Woodbury ME, Vermeiren Y, Lambourne M, et al. Distinct amyloid-β and tau-associated microglia profiles in Alzheimer’s disease. Acta Neuropathol (Berl). 2021;141:681–96. 10.1007/s00401-021-02263-w. PMC8043951 33609158

[12] Millet A, Ledo JH, Tavazoie S. An Exhausted-Like Microglial Population Accumulates in Aged and APOE4 Genotype Alzheimer’s Brains. Immunity. 2024;57:153–e1706. 10.1016/j.immuni.2023.12.001. PMC10805152 38159571

[13] Hamelin L, Lagarde J, Dorothée G, Leroy C, Labit M, Comley RA, et al. Early and protective microglial activation in Alzheimer’s disease: a prospective study using 18F-DPA-714 PET imaging. Brain J Neurol. 2016;139:1252–64. 10.1093/brain/aww017. 26984188

[14] Morenas-Rodríguez E, Li Y, Nuscher B, Franzmeier N, Xiong C, Suárez-Calvet M, et al. Soluble TREM2 in CSF and its association with other biomarkers and cognition in autosomal-dominant Alzheimer’s disease: a longitudinal observational study. Lancet Neurol. 2022;21:329–41. 10.1016/S1474-4422(22)00027-8. PMC8926925 35305339

[15] Liddelow SA, Guttenplan KA, Clarke LE, Bennett FC, Bohlen CJ, Schirmer L, et al. Neurotoxic reactive astrocytes are induced by activated microglia. Nature. 2017;541:481–7. 10.1038/nature21029. PMC5404890 28099414

[16] Chun H, Im H, Kang YJ, Kim Y, Shin JH, Won W, et al. Severe reactive astrocytes precipitate pathological hallmarks of Alzheimer’s disease via H2O2 – production. Nat Neurosci. 2020;23:1555–66. 10.1038/s41593-020-00735-y. 33199896

[17] Maphis N, Xu G, Kokiko-Cochran ON, Jiang S, Cardona A, Ransohoff RM, et al. Reactive microglia drive tau pathology and contribute to the spreading of pathological tau in the brain. Brain. 2015;138:1738–55. 10.1093/brain/awv081. PMC4542622 25833819

[18] Huang Y, Happonen KE, Burrola PG, O’Connor C, Hah N, Huang L, et al. Microglia use TAM receptors to detect and engulf amyloid β plaques. Nat Immunol. 2021;22:586–94. 10.1038/s41590-021-00913-5. PMC8102389 33859405

[19] Baligács N, Albertini G, Borrie SC, Serneels L, Pridans C, Balusu S, et al. Homeostatic microglia initially seed and activated microglia later reshape amyloid plaques in Alzheimer’s Disease. Nat Commun. 2024;15:10634. 10.1038/s41467-024-54779-w. PMC11621353 39639016

[20] Spangenberg E, Severson PL, Hohsfield LA, Crapser J, Zhang J, Burton EA, et al. Sustained microglial depletion with CSF1R inhibitor impairs parenchymal plaque development in an Alzheimer’s disease model. Nat Commun. 2019;10:3758. 10.1038/s41467-019-11674-z. PMC6704256 31434879

[21] Edison P, Brooks DJ. Role of Neuroinflammation in the Trajectory of Alzheimer’s Disease and in vivo Quantification Using PET. J Alzheimers Dis JAD. 2018;64:S339–51. 10.3233/JAD-179929. 29865053

[22] Dani M, Wood M, Mizoguchi R, Fan Z, Walker Z, Morgan R, et al. Microglial activation correlates in vivo with both tau and amyloid in Alzheimer’s disease. Brain J Neurol. 2018;141:2740–54. 10.1093/brain/awy188. 30052812

[23] Kreisl WC, Lyoo CH, McGwier M, Snow J, Jenko KJ, Kimura N, et al. In vivo radioligand binding to translocator protein correlates with severity of Alzheimer’s disease. Brain J Neurol. 2013;136:2228–38. 10.1093/brain/awt145. PMC3692038 23775979

[24] Parbo P, Ismail R, Hansen KV, Amidi A, Mårup FH, Gottrup H, et al. Brain inflammation accompanies amyloid in the majority of mild cognitive impairment cases due to Alzheimer’s disease. Brain. 2017;140:2002–11. 10.1093/brain/awx120. 28575151

[25] Hamelin L, Lagarde J, Dorothée G, Potier MC, Corlier F, Kuhnast B, et al. Distinct dynamic profiles of microglial activation are associated with progression of Alzheimer’s disease. Brain J Neurol. 2018;141:1855–70. 10.1093/brain/awy079. 29608645

[26] Fan Z, Brooks DJ, Okello A, Edison P. An early and late peak in microglial activation in Alzheimer’s disease trajectory. Brain J Neurol. 2017;140:792–803. 10.1093/brain/aww349. PMC5837520 28122877

[27] Femminella GD, Dani M, Wood M, Fan Z, Calsolaro V, Atkinson R, et al. Microglial activation in early Alzheimer trajectory is associated with higher gray matter volume. Neurology. 2019;92:e1331–43. 10.1212/WNL.0000000000007133. PMC6511099 30796139

[28] Franzmeier N, Dewenter A, Frontzkowski L, Dichgans M, Rubinski A, Neitzel J, et al. Patient-centered connectivity-based prediction of tau pathology spread in Alzheimer’s disease. Sci Adv. 2020;6:eabd1327. 10.1126/sciadv.abd1327. PMC7695466 33246962

[29] Franzmeier N, Neitzel J, Rubinski A, Smith R, Strandberg O, Ossenkoppele R, et al. Functional brain architecture is associated with the rate of tau accumulation in Alzheimer’s disease. Nat Commun. 2020;11:347. 10.1038/s41467-019-14159-1. PMC6969065 31953405

[30] Rauchmann B, Brendel M, Franzmeier N, Trappmann L, Zaganjori M, Ersoezlue E, et al. Microglial Activation and Connectivity in Alzheimer Disease and Aging. Ann Neurol. 2022;92:768–81. 10.1002/ana.26465. 36053756

[31] Malpetti M, Roemer SN, Harris S, Gross M, Gnörich J, Stephens A, et al. Neuroinflammation Parallels 18F-PI-2620 Positron Emission Tomography Patterns in Primary 4-Repeat Tauopathies. Mov Disord. 2024;39:1480–92. 10.1002/mds.29924. 39022835

[32] Finze A, Biechele G, Rauchmann B-S, Franzmeier N, Palleis C, Katzdobler S, et al. Individual regional associations between Aβ-, tau- and neurodegeneration (ATN) with microglial activation in patients with primary and secondary tauopathies. Mol Psychiatry. 2023;28:4438–50. 10.1038/s41380-023-02188-8. PMC10827660 37495886

[33] Rauchmann B, Brendel M, Keeser D, Tato M, Palleis C, Zaganjori M, et al. Microglial activation and brain networks in Alzheimer’s disease: The ActiGliA cohort study: Neuroimaging / Multi-modal comparisons. Alzheimers Dement. 2020;16:e043265. 10.1002/alz.043265.

[34] Kreisl WC, Jenko KJ, Hines CS, Lyoo CH, Corona W, Morse CL, et al. A Genetic Polymorphism for Translocator Protein 18 Kda Affects both in Vitro and in Vivo Radioligand Binding in Human Brain to this Putative Biomarker of Neuroinflammation. J Cereb Blood Flow Metab. 2013;33:53–8. 10.1038/jcbfm.2012.131. PMC3597372 22968319

[35] Avants BB, Tustison NJ, Song G, Cook PA, Klein A, Gee JC. A reproducible evaluation of ANTs similarity metric performance in brain image registration. NeuroImage. 2011;54:2033–44. 10.1016/j.neuroimage.2010.09.025. PMC3065962 20851191

[36] Jenkinson M, Beckmann CF, Behrens TEJ, Woolrich MW, Smith SM. FSL NeuroImage. 2012;62:782–90. 10.1016/j.neuroimage.2011.09.015. 21979382

[37] Schaefer A, Kong R, Gordon EM, Laumann TO, Zuo X-N, Holmes AJ, et al. Local-global parcellation of the human cerebral cortex from intrinsic functional connectivity MRI. Cereb Cortex. 2018;28:3095–114. 10.1093/cercor/bhx179. PMC6095216 28981612

[38] Vos de Wael R, Benkarim O, Paquola C, Larivière S, Royer J, Tavakol S, et al. BrainSpace: a toolbox for the analysis of macroscale gradients in neuroimaging and connectomics datasets. Commun Biol. 2020;3:103. 10.1038/s42003-020-0794-7. PMC7058611 32139786

[39] Esteban O, Markiewicz CJ, Blair RW, Moodie CA, Isik AI, Erramuzpe A, et al. fMRIPrep: a robust preprocessing pipeline for functional MRI. Nat Methods. 2019;16:111–6. 10.1038/s41592-018-0235-4. PMC6319393 30532080

[40] Gorgolewski K, Burns CD, Madison C, Clark D, Halchenko YO, Waskom ML, et al. Nipype: a flexible, lightweight and extensible neuroimaging data processing framework in Python. Front Neuroinform. 2011;5:13. 10.3389/fninf.2011.00013. PMC3159964 21897815

[41] Dale AM, Fischl B, Sereno MI. Cortical surface-based analysis. I. Segmentation and surface reconstruction. NeuroImage. 1999;9:179–94. 10.1006/nimg.1998.0395. 9931268

[42] Abraham A, Pedregosa F, Eickenberg M, Gervais P, Mueller A, Kossaifi J, et al. Machine learning for neuroimaging with scikit-learn. Front Neuroinform. 2014;8:14. 10.3389/fninf.2014.00014. PMC3930868 24600388

[43] Harris CR, Millman KJ, van der Walt SJ, Gommers R, Virtanen P, Cournapeau D, et al. Array programming with NumPy. Nature. 2020;585:357–62. 10.1038/s41586-020-2649-2. PMC7759461 32939066

[44] McKinney W. Data Structures for Statistical Computing in Python. In: Proceedings of the 9th Python in Science Conference. 2010. pp. 56–61. 10.25080/Majora-92bf1922-00a.

[45] Virtanen P, Gommers R, Oliphant TE, Haberland M, Reddy T, Cournapeau D, et al. SciPy 1.0: fundamental algorithms for scientific computing in Python. Nat Methods. 2020;17:261–72. 10.1038/s41592-019-0686-2. PMC7056644 32015543

[46] Seabold S, Perktold J, Statsmodels. Econometric and Statistical Modeling with Python. In: Proceedings of the 9th Python in Science Conference. 2010. pp. 92 – 6. 10.25080/Majora-92bf1922-011.

[47] Crook H, Wahdan M, Tuil ME, Livingston NR, Raza S, Nowell J, et al. CSF sTREM2 is associated with neuroprotective microglial states early in Alzheimer’s disease and deleterious effects later in the disease trajectory. Alzheimers Dement. 2024;20:e092410.

[48] Albrecht DS, Sagare A, Pachicano M, Sweeney MD, Toga A, Zlokovic B, et al. Early neuroinflammation is associated with lower amyloid and tau levels in cognitively normal older adults. Brain Behav Immun. 2021;94:299–307. 10.1016/j.bbi.2021.01.010. PMC8793040 33486003

[49] Sebastian Monasor L, Müller SA, Colombo AV, Tanrioever G, König J, Roth S, et al. Fibrillar Aβ triggers microglial proteome alterations and dysfunction in Alzheimer mouse models. eLife. 2020;9:e54083. 10.7554/eLife.54083. PMC7279888 32510331

[50] Swanson MEV, Scotter EL, Smyth LCD, Murray HC, Ryan B, Turner C, et al. Identification of a dysfunctional microglial population in human Alzheimer’s disease cortex using novel single-cell histology image analysis. Acta Neuropathol Commun. 2020;8:170. 10.1186/s40478-020-01047-9. PMC7576851 33081847

[51] Hopp SC, Lin Y, Oakley D, Roe AD, DeVos SL, Hanlon D, et al. The role of microglia in processing and spreading of bioactive tau seeds in Alzheimer’s disease. J Neuroinflammation. 2018;15:269. 10.1186/s12974-018-1309-z. PMC6145371 30227881

[52] Beckman D, Diniz GB, Ott S, Hobson B, Chaudhari AJ, Muller S, et al. Temporal progression of tau pathology and neuroinflammation in a rhesus monkey model of Alzheimer’s disease. Alzheimers Dement. 2024;20:5198–219. 10.1002/alz.13868. PMC11350056 39030748

[53] Sanchez-Mejias E, Navarro V, Jimenez S, Sanchez-Mico M, Sanchez-Varo R, Nuñez-Diaz C, et al. Soluble phospho-tau from Alzheimer’s disease hippocampus drives microglial degeneration. Acta Neuropathol (Berl). 2016;132:897–916. 10.1007/s00401-016-1630-5. PMC5106501 27743026

[54] Karabag D, Scheiblich H, Griep A, Santarelli F, Schwartz S, Heneka MT, et al. Characterizing microglial senescence: Tau as a key player. J Neurochem. 2023;166:517–33. 10.1111/jnc.15866. 37278117

[55] Bolós M, Llorens-Martín M, Jurado-Arjona J, Hernández F, Rábano A, Avila J. Direct Evidence of Internalization of Tau by Microglia In Vitro and In Vivo. J Alzheimer’s Dis. 2016;50:77–87. 10.3233/JAD-150704. 26638867

[56] Mandrekar S, Jiang Q, Lee CYD, Koenigsknecht-Talboo J, Holtzman DM, Landreth GE. Microglia mediate the clearance of soluble Abeta through fluid phase macropinocytosis. J Neurosci Off J Soc Neurosci. 2009;29:4252–62. 10.1523/JNEUROSCI.5572-08.2009. PMC3034143 19339619

[57] Wang C, Fan L, Khawaja RR, Liu B, Zhan L, Kodama L, et al. Microglial NF-κB drives tau spreading and toxicity in a mouse model of tauopathy. Nat Commun. 2022;13:1969. 10.1038/s41467-022-29552-6. PMC9005658 35413950

[58] Streit WJ, Braak H, Xue Q-S, Bechmann I. Dystrophic (senescent) rather than activated microglial cells are associated with tau pathology and likely precede neurodegeneration in Alzheimer’s disease. Acta Neuropathol (Berl). 2009;118:475–85. 10.1007/s00401-009-0556-6. PMC2737117 19513731

[59] Vogel JW, Young AL, Oxtoby NP, Smith R, Ossenkoppele R, Strandberg OT, et al. Four distinct trajectories of tau deposition identified in Alzheimer’s disease. Nat Med. 2021;27:871–81. 10.1038/s41591-021-01309-6. PMC8686688 33927414

[60] Shojaei M, Schaefer R, Schlepckow K, Kunze LH, Struebing FL, Brunner B, et al. PET imaging of microglia in Alzheimer’s disease using copper-64 labeled TREM2 antibodies. Theranostics. 2024;14:6319–36. 10.7150/thno.97149. PMC11488106 39431020

[61] Joseph E, Kunze LH, Schaefer R, Palumbo G, Kugelmann B, Wagner S, et al. Design, Synthesis and Preclinical Evaluation of a Brain-Permeable PET Tracer for P2Y12 Receptor Imaging in the Brain. J Med Chem. 2025;68:15543–62. 10.1021/acs.jmedchem.5c00457. PMC12362594 40713021

