# Supplementary material for: Microglial dynamics associate with the spatial pattern of tau deposition in early Alzheimer’s disease
Source: Alzheimers Res Ther. 2026 Oct 12;18:232. doi: 10.1186/s13195-026-02230-1 (PMC13663006; doi:10.1186/s13195-026-02230-1)
Supplement: Supplementary file 1 [file 13195_2026_2230_MOESM1_ESM.docx]

# **Supplementary Materials**

# **Table S1: Demographics and clinical data for participants with TSPO PET at baseline and follow-up**

| **Characteristic** | **ADS (n = 5)** | **HC (n = 11)** | **p** |
| --- | --- | --- | --- |
| Age (years) | 74.0 [73.0, 79.0] | 72.0 [68.5, 75.0] | 0.426 |
| Sex (F) | 3 (60.0%) | 6 (54.5%) | 1.0 |
| Weight (kg) | 62.0 [55.0, 73.0] | 78.0 [60.0, 85.5] | 0.124 |
| Education (years) | 12.0 [11.0, 19.0] | 15.0 [12.5, 18.0] | 0.776 |
| MMSE | 25.0 [21.0, 26.0] | 30.0 [29.0, 30.0] | 0.001 |
| CDR-SOB | 3.0 [2.5, 3.0] | 0.0 [0.0, 0.0] | <0.001 |
| Global CDR | 0.5 [0.5, 0.5] | 0.0 [0.0, 0.0] | <0.001 |
| CERAD-NB | 56.5 [53.0, 71.5] | 88.0 [85.5, 90.5] | 0.002 |
| CSF Aβ42 (pg/mL) | 356.9 [300.5, 388.3] | 1148.0 [531.4, 1422.5] | 0.040 |
| CSF Aβ42/40 ratio | 0.03 [0.03, 0.04] | 0.07 [0.07, 0.08] | 0.001 |
| CSF p-tau181 (pg/mL) | 91.5 [78.2, 98.0] | 52.4 [42.5, 71.5] | 0.026 |
| CSF t-tau (pg/mL) | 536.3 [344.3, 683.5] | 256.4 [196.7, 352.8] | 0.177 |
| APOE ε4 carriers (%) | 3 (60%) | 2 (18.2%) | 0.177 |
| rs6971 LAB status (%) | 0 (0%) | 0 (0%) | 1.0 |

# **Table S2: Epicenter Threshold Sensitivity**

**ADS: log(Tau) ~ FC to TSPO change epicenters + FC to TSPO BL epicenters**

| **Term** | **Statistic** | **5%** | **10%** | **15%** | **20%** |
| --- | --- | --- | --- | --- | --- |
| FC to TSPO change epicenter | β | −0.357 | −0.409 | −0.452 | −0.543 |
|  | exp(β) | 0.700 | 0.664 | 0.637 | 0.581 |
|  | p | <0.001 | <0.001 | <0.001 | <0.001 |
| FC to TSPO BL epicenter | β | 0.337 | 0.348 | 0.373 | 0.308 |
|  | exp(β) | 1.401 | 1.417 | 1.452 | 1.361 |
|  | p | <0.001 | <0.001 | <0.001 | <0.001 |

**HC: log(Tau) ~ FC to TSPO change epicenters + FC to TSPO BL epicenters**

| **Term** | **Statistic** | **5%** | **10%** | **15%** | **20%** |
| --- | --- | --- | --- | --- | --- |
| FC to TSPO change epicenter | β | −0.025 | −0.017 | −0.007 | −0.038 |
|  | exp(β) | 0.975 | 0.983 | 0.992 | 0.963 |
|  | p | 0.265 | 0.521 | 0.830 | 0.290 |
| FC to TSPO BL epicenter | β | 0.127 | 0.204 | 0.317 | 0.447 |
|  | exp(β) | 1.136 | 1.227 | 1.373 | 1.564 |
|  | p | <0.001 | <0.001 | <0.001 | <0.001 |

# **Table S3: Out-of-Sample Predictive Validation (LME)**

| **Model** | **Test set** | **n** | **Term** | **β [95% CI]** | **exp(β) [95% CI]** | **p** |
| --- | --- | --- | --- | --- | --- | --- |
| Single | ADS | 10 | FC to TSPO BL epicenter | 0.449 [0.30, 0.60] | 1.57 [1.34, 1.83] | <0.001 |
|  | ADS | 10 | FC to TSPO change epicenter | −0.518 [−0.70, −0.34] | 0.60 [0.50, 0.71] | <0.001 |
| Single | HC | 13 | FC to TSPO BL epicenter | 0.026 [−0.02, 0.07] | 1.03 [0.98, 1.08] | 0.269 |
|  | HC | 13 | FC to TSPO change epicenter | −0.124 [−0.19, −0.06] | 0.88 [0.83, 0.94] | <0.001 |
| Single | ADS (indep) | 5 | FC to TSPO change epicenter | −0.647 [−0.90, −0.40] | 0.52 [0.41, 0.67] | <0.001 |
| Single | ADS (indep) | 5 | FC to TSPO BL epicenter | 0.589 [0.37, 0.81] | 1.80 [1.45, 2.25] | <0.001 |
| Combined | ADS (indep) | 5 | FC to TSPO BL epicenter | 0.549 [0.28, 0.82] | 1.73 [1.33, 2.26] | <0.001 |
|  |  | 5 | FC to TSPO change epicenter | −0.326 [−0.61, −0.04] | 0.72 [0.54, 0.96] | 0.026 |
| Combined | ADS (all) | 10 | FC to TSPO BL epicenter | 0.390 [0.20, 0.58] | 1.48 [1.22, 1.78] | <0.001 |
|  |  | 10 | FC to TSPO change epicenter | −0.338 [−0.55, −0.13] | 0.71 [0.58, 0.88] | 0.001 |

# **Table S4: TSPO Change ~ Network × Baseline TSPO**

**ADS (n = 5):**

| **Interaction term** | **β [95% CI]** | **exp(β) [95% CI]** | **p** | **q(FDR)** |
| --- | --- | --- | --- | --- |
| Main: log(TSPO BL) | −0.177 [−0.25, −0.10] | 0.838 [0.78, 0.90] | <0.001 | — |
| Limbic × BL | 0.107 [−0.01, 0.22] | 1.113 [0.99, 1.24] | 0.062 | 0.131 |
| SalVentAttn × BL | −0.117 [−0.24, 0.01] | 0.890 [0.79, 1.01] | 0.066 | 0.131 |
| Vis × BL | 0.119 [0.03, 0.21] | 1.126 [1.03, 1.23] | 0.007 | 0.042 |
| Default × BL | −0.031 [−0.13, 0.06] | 0.969 [0.88, 1.07] | 0.524 | 0.524 |
| DorsAttn × BL | 0.042 [−0.05, 0.13] | 1.043 [0.95, 1.14] | 0.371 | 0.524 |
| SomMot × BL | −0.035 [−0.14, 0.07] | 0.965 [0.87, 1.07] | 0.514 | 0.524 |

**HC (n = 11)**

| **Interaction term** | **β [95% CI]** | **exp(β) [95% CI]** | **p** | **q(FDR)** |
| --- | --- | --- | --- | --- |
| Main: log(TSPO_BL) | −0.394 [−0.48, −0.31] | 0.674 [0.62, 0.73] | <0.001 | — |
| Limbic × BL | −0.054 [−0.17, 0.07] | 0.947 [0.84, 1.07] | 0.379 | 0.568 |
| SalVentAttn × BL | 0.085 [−0.06, 0.23] | 1.089 [0.95, 1.25] | 0.234 | 0.469 |
| DorsAttn × BL | 0.240 [0.14, 0.34] | 1.271 [1.15, 1.41] | <0.001 | <0.001 |
| Default × BL | 0.031 [−0.07, 0.13] | 1.032 [0.94, 1.14] | 0.529 | 0.635 |
| SomMot × BL | −0.024 [−0.13, 0.08] | 0.976 [0.88, 1.08] | 0.648 | 0.648 |
| Vis × BL | 0.236 [0.15, 0.32] | 1.266 [1.16, 1.38] | <0.001 | <0.001 |

# **Table S5: TSPO Change ~ Braak Stage × Baseline TSPO**

**ADS (n = 5): TSPO LogRatio ~ C(Braak) × log(TSPO_BL)**

| **Interaction term** | **β [95% CI]** | **exp(β) [95% CI]** | **p** | **q(FDR)** |
| --- | --- | --- | --- | --- |
| Main: log(TSPO_BL) | −0.658 [−1.04, −0.27] | 0.518 [0.35, 0.76] | <0.001 | — |
| Braak III × BL | 0.607 [0.21, 1.00] | 1.834 [1.24, 2.72] | 0.002 | 0.009 |
| Braak IV × BL | 0.524 [0.13, 0.92] | 1.689 [1.14, 2.50] | 0.009 | 0.009 |
| Braak V × BL | 0.516 [0.13, 0.90] | 1.675 [1.14, 2.47] | 0.009 | 0.009 |
| Braak VI × BL | 0.539 [0.15, 0.93] | 1.715 [1.16, 2.53] | 0.007 | 0.009 |

**HC (n = 11): TSPO LogRatio ~ C(Braak) × log(TSPO_BL)**

| **Interaction term** | **β [95% CI]** | **exp(β) [95% CI]** | **p** | **q(FDR)** |
| --- | --- | --- | --- | --- |
| Main: log(TSPO_BL) | −0.612 [−0.82, −0.41] | 0.542 [0.44, 0.67] | <0.001 | — |
| Braak III × BL | 0.439 [0.22, 0.66] | 1.551 [1.25, 1.93] | <0.001 | <0.001 |
| Braak IV × BL | 0.378 [0.15, 0.60] | 1.459 [1.17, 1.83] | <0.001 | 0.002 |
| Braak V × BL | 0.338 [0.13, 0.55] | 1.402 [1.14, 1.73] | 0.002 | 0.002 |
| Braak VI × BL | 0.334 [0.12, 0.55] | 1.396 [1.12, 1.73] | 0.003 | 0.003 |

# **Table S6: Follow-up Tau ~ Baseline TSPO × Network**

**ADS (n = 5): log(Tau) ~ log(TSPO_BL) × C(Network)**

| **Interaction term** | **β [95% CI]** | **exp(β) [95% CI]** | **p** | **q(FDR)** |
| --- | --- | --- | --- | --- |
| Main: log(TSPO_BL) | 0.466 [0.26, 0.68] | 1.593 [1.29, 1.97] | <0.001 | — |
| Limbic × BL | 0.396 [0.07, 0.72] | 1.486 [1.07, 2.06] | 0.018 | 0.106 |
| Default × BL | 0.239 [−0.03, 0.51] | 1.270 [0.97, 1.66] | 0.081 | 0.163 |
| DorsAttn × BL | −0.197 [−0.47, 0.08] | 0.821 [0.63, 1.08] | 0.158 | 0.237 |
| SalVentAttn × BL | 0.300 [−0.02, 0.62] | 1.350 [0.98, 1.87] | 0.069 | 0.163 |
| SomMot × BL | 0.065 [−0.23, 0.36] | 1.067 [0.80, 1.43] | 0.660 | 0.660 |
| Vis × BL | −0.163 [−0.41, 0.09] | 0.850 [0.66, 1.09] | 0.202 | 0.242 |

**HC (n = 11): log(Tau) ~ log(TSPO_BL) × C(Network)**

| **Interaction term** | **β [95% CI]** | **exp(β) [95% CI]** | **p** | **q(FDR)** |
| --- | --- | --- | --- | --- |
| Main: log(TSPO_BL) | 0.459 [0.40, 0.52] | 1.583 [1.49, 1.68] | <0.001 | — |
| DorsAttn × BL | −0.038 [−0.12, 0.04] | 0.963 [0.89, 1.04] | 0.354 | 0.531 |
| Limbic × BL | −0.071 [−0.16, 0.02] | 0.932 [0.85, 1.02] | 0.141 | 0.282 |
| SomMot × BL | −0.077 [−0.16, 0.00] | 0.926 [0.86, 1.00] | 0.059 | 0.176 |
| Vis × BL | −0.166 [−0.24, −0.10] | 0.847 [0.79, 0.91] | <0.001 | <0.001 |
| Default × BL | 0.022 [−0.05, 0.10] | 1.022 [0.95, 1.10] | 0.572 | 0.687 |
| SalVentAttn × BL | −0.012 [−0.12, 0.09] | 0.988 [0.89, 1.10] | 0.826 | 0.826 |

# **Table S7: Tau ~ Baseline TSPO × Braak Stage**

**ADS (n = 5):**

| **Interaction term** | **β [95% CI]** | **exp(β) [95% CI]** | **p** | **q(FDR)** |
| --- | --- | --- | --- | --- |
| Main: log(TSPO_BL) | −0.496 [−1.44, 0.45] | 0.609 [0.24, 1.56] | 0.302 | — |
| Braak III × BL | 0.879 [−0.09, 1.85] | 2.409 [0.91, 6.34] | 0.075 | 0.075 |
| Braak IV × BL | 1.310 [0.35, 2.27] | 3.708 [1.42, 9.67] | 0.007 | 0.030 |
| Braak V × BL | 0.991 [0.04, 1.94] | 2.694 [1.05, 6.95] | 0.040 | 0.073 |
| Braak VI × BL | 0.938 [−0.02, 1.90] | 2.556 [0.98, 6.66] | 0.055 | 0.073 |

**HC (n = 11):**

| **Interaction term** | **β [95% CI]** | **exp(β) [95% CI]** | **p** | **q(FDR)** |
| --- | --- | --- | --- | --- |
| Main: log(TSPO_BL) | 0.342 [0.19, 0.49] | 1.408 [1.21, 1.63] | <0.001 | — |
| Braak III × BL | −0.113 [−0.27, 0.05] | 0.894 [0.76, 1.05] | 0.163 | 0.526 |
| Braak IV × BL | 0.033 [−0.13, 0.20] | 1.034 [0.88, 1.22] | 0.687 | 0.687 |
| Braak V × BL | 0.086 [−0.06, 0.24] | 1.090 [0.94, 1.27] | 0.263 | 0.526 |
| Braak VI × BL | 0.046 [−0.11, 0.20] | 1.047 [0.90, 1.22] | 0.561 | 0.687 |

**Table S8: TSPO baseline epicenters**

| **ADS** | | **HC** | |
| --- | --- | --- | --- |
| Schaefer ROI | Yeo7 Network | Schaefer ROI | Yeo7 Network |
| 7Networks_LH_Cont_Temp_1 | Control | 7Networks_LH_Cont_OFC_1 | Control |
| 7Networks_RH_Cont_Cing_1 | Control | 7Networks_LH_Cont_Temp_1 | Control |
| 7Networks_RH_Cont_Temp_1 | Control | 7Networks_RH_Cont_PFCl_1 | Control |
| 7Networks_LH_Default_PFC_3 | Default | 7Networks_LH_Default_PFC_3 | Default |
| 7Networks_LH_Default_Par_2 | Default | 7Networks_LH_Default_Temp_1 | Default |
| 7Networks_LH_Default_Temp_1 | Default | 7Networks_LH_Default_Temp_2 | Default |
| 7Networks_LH_Default_Temp_2 | Default | 7Networks_LH_Default_pCunPCC_2 | Default |
| 7Networks_LH_Default_pCunPCC_2 | Default | 7Networks_LH_Default_pCunPCC_3 | Default |
| 7Networks_LH_Default_pCunPCC_3 | Default | 7Networks_RH_Default_Temp_1 | Default |
| 7Networks_RH_Default_pCunPCC_2 | Default | 7Networks_RH_Default_pCunPCC_2 | Default |
| 7Networks_LH_DorsAttn_Post_1 | DorsAttn | 7Networks_LH_DorsAttn_Post_1 | DorsAttn |
| 7Networks_LH_DorsAttn_Post_2 | DorsAttn | 7Networks_LH_DorsAttn_Post_2 | DorsAttn |
| 7Networks_LH_DorsAttn_Post_9 | DorsAttn | 7Networks_LH_DorsAttn_Post_9 | DorsAttn |
| 7Networks_RH_DorsAttn_Post_1 | DorsAttn | 7Networks_RH_DorsAttn_Post_1 | DorsAttn |
| 7Networks_LH_Limbic_OFC_1 | Limbic | 7Networks_RH_DorsAttn_Post_6 | DorsAttn |
| 7Networks_LH_Limbic_TempPole_1 | Limbic | 7Networks_LH_Limbic_OFC_1 | Limbic |
| 7Networks_LH_Limbic_TempPole_2 | Limbic | 7Networks_LH_Limbic_OFC_2 | Limbic |
| 7Networks_LH_Limbic_TempPole_3 | Limbic | 7Networks_LH_Limbic_TempPole_1 | Limbic |
| 7Networks_RH_Limbic_OFC_1 | Limbic | 7Networks_LH_Limbic_TempPole_3 | Limbic |
| 7Networks_RH_Limbic_OFC_2 | Limbic | 7Networks_RH_Limbic_OFC_1 | Limbic |
| 7Networks_RH_Limbic_TempPole_1 | Limbic | 7Networks_RH_Limbic_OFC_2 | Limbic |
| 7Networks_RH_Limbic_TempPole_2 | Limbic | 7Networks_RH_Limbic_TempPole_1 | Limbic |
| 7Networks_RH_Limbic_TempPole_3 | Limbic | 7Networks_RH_Limbic_TempPole_2 | Limbic |
| 7Networks_LH_Vis_11 | Vis | 7Networks_RH_Limbic_TempPole_3 | Limbic |
| 7Networks_LH_Vis_13 | Vis | 7Networks_LH_SomMot_15 | SomMot |
| 7Networks_LH_Vis_2 | Vis | 7Networks_LH_Vis_13 | Vis |
| 7Networks_LH_Vis_3 | Vis | 7Networks_LH_Vis_2 | Vis |
| 7Networks_LH_Vis_4 | Vis | 7Networks_LH_Vis_3 | Vis |
| 7Networks_LH_Vis_5 | Vis | 7Networks_LH_Vis_4 | Vis |
| 7Networks_LH_Vis_7 | Vis | 7Networks_LH_Vis_5 | Vis |
| 7Networks_LH_Vis_9 | Vis | 7Networks_LH_Vis_7 | Vis |
| 7Networks_RH_Vis_1 | Vis | 7Networks_RH_Vis_1 | Vis |
| 7Networks_RH_Vis_12 | Vis | 7Networks_RH_Vis_12 | Vis |
| 7Networks_RH_Vis_14 | Vis | 7Networks_RH_Vis_14 | Vis |
| 7Networks_RH_Vis_3 | Vis | 7Networks_RH_Vis_3 | Vis |
| 7Networks_RH_Vis_4 | Vis | 7Networks_RH_Vis_4 | Vis |
| 7Networks_RH_Vis_5 | Vis | 7Networks_RH_Vis_5 | Vis |
| 7Networks_RH_Vis_6 | Vis | 7Networks_RH_Vis_6 | Vis |
| 7Networks_RH_Vis_8 | Vis | 7Networks_RH_Vis_8 | Vis |
| 7Networks_RH_Vis_9 | Vis | 7Networks_RH_Vis_9 | Vis |

**Table S9: TSPO change epicenters**

| **ADS** | | **HC** | |
| --- | --- | --- | --- |
| Schaefer ROI | Yeo7 Network | Schaefer ROI | Yeo7 Network |
| 7Networks_LH_Cont_PFCl_1 | Control | 7Networks_LH_Cont_PFCl_1 | Control |
| 7Networks_LH_Cont_PFCl_3 | Control | 7Networks_LH_Cont_Temp_1 | Control |
| 7Networks_LH_Cont_PFCl_5 | Control | 7Networks_RH_Cont_PFCmp_2 | Control |
| 7Networks_LH_Cont_Par_3 | Control | 7Networks_RH_Cont_Par_1 | Control |
| 7Networks_RH_Cont_PFCl_1 | Control | 7Networks_RH_Cont_Temp_1 | Control |
| 7Networks_RH_Cont_PFCl_2 | Control | 7Networks_LH_Default_PFC_13 | Default |
| 7Networks_RH_Cont_PFCl_5 | Control | 7Networks_LH_Default_PFC_9 | Default |
| 7Networks_RH_Cont_pCun_1 | Control | 7Networks_LH_Default_Par_1 | Default |
| 7Networks_LH_Default_PFC_11 | Default | 7Networks_LH_Default_Par_3 | Default |
| 7Networks_LH_Default_PFC_3 | Default | 7Networks_LH_Default_Temp_4 | Default |
| 7Networks_LH_Default_PFC_4 | Default | 7Networks_LH_Default_Temp_5 | Default |
| 7Networks_LH_Default_PFC_5 | Default | 7Networks_LH_Default_pCunPCC_1 | Default |
| 7Networks_LH_Default_PFC_7 | Default | 7Networks_RH_Default_PFCdPFCm_2 | Default |
| 7Networks_LH_Default_PFC_9 | Default | 7Networks_RH_Default_PFCdPFCm_4 | Default |
| 7Networks_RH_Default_PFCdPFCm_5 | Default | 7Networks_RH_Default_PFCdPFCm_5 | Default |
| 7Networks_RH_Default_PFCdPFCm_6 | Default | 7Networks_RH_Default_Temp_5 | Default |
| 7Networks_RH_Default_PFCdPFCm_7 | Default | 7Networks_LH_DorsAttn_Post_2 | DorsAttn |
| 7Networks_RH_Default_Temp_5 | Default | 7Networks_LH_DorsAttn_Post_4 | DorsAttn |
| 7Networks_LH_DorsAttn_FEF_1 | DorsAttn | 7Networks_LH_SalVentAttn_ParOper_1 | DorsAttn |
| 7Networks_LH_DorsAttn_FEF_2 | DorsAttn | 7Networks_RH_DorsAttn_Post_1 | DorsAttn |
| 7Networks_LH_DorsAttn_Post_1 | DorsAttn | 7Networks_RH_DorsAttn_Post_2 | DorsAttn |
| 7Networks_LH_DorsAttn_Post_5 | DorsAttn | 7Networks_LH_Limbic_TempPole_4 | Limbic |
| 7Networks_LH_Limbic_OFC_1 | Limbic | 7Networks_LH_SalVentAttn_Med_2 | SalVentAttn |
| 7Networks_RH_Limbic_OFC_1 | Limbic | 7Networks_LH_SomMot_14 | SomMot |
| 7Networks_RH_Limbic_TempPole_3 | Limbic | 7Networks_RH_SomMot_8 | SomMot |
| 7Networks_LH_SalVentAttn_Med_2 | SalVentAttn | 7Networks_LH_Vis_1 | Vis |
| 7Networks_LH_SalVentAttn_ParOper_3 | SalVentAttn | 7Networks_LH_Vis_11 | Vis |
| 7Networks_RH_SalVentAttn_FrOperIns_3 | SalVentAttn | 7Networks_LH_Vis_2 | Vis |
| 7Networks_RH_SalVentAttn_Med_3 | SalVentAttn | 7Networks_LH_Vis_3 | Vis |
| 7Networks_LH_SomMot_12 | SomMot | 7Networks_LH_Vis_5 | Vis |
| 7Networks_LH_SomMot_2 | SomMot | 7Networks_LH_Vis_7 | Vis |
| 7Networks_LH_SomMot_8 | SomMot | 7Networks_LH_Vis_8 | Vis |
| 7Networks_LH_SomMot_9 | SomMot | 7Networks_RH_Vis_1 | Vis |
| 7Networks_RH_SomMot_11 | SomMot | 7Networks_RH_Vis_11 | Vis |
| 7Networks_RH_SomMot_14 | SomMot | 7Networks_RH_Vis_12 | Vis |
| 7Networks_RH_SomMot_15 | SomMot | 7Networks_RH_Vis_15 | Vis |
| 7Networks_RH_SomMot_17 | SomMot | 7Networks_RH_Vis_3 | Vis |
| 7Networks_RH_SomMot_19 | SomMot | 7Networks_RH_Vis_5 | Vis |
| 7Networks_RH_SomMot_7 | SomMot | 7Networks_RH_Vis_6 | Vis |
| 7Networks_LH_Vis_5 | Vis | 7Networks_RH_Vis_9 | Vis |

**Table S10: Sensitivity analyses for APOε4 status and TSPO binding**

| **Model / effect** | **Unadjusted** | **+ APOE ε4** | **+ HAB/MAB** | **+ HAB/MAB + APOE ε4** |
| --- | --- | --- | --- | --- |
| log(tau) ~ log(BL TSPO) [ADS] | 1.72 [1.60, 1.86] | 1.72 [1.60, 1.86] | 1.72 [1.60, 1.86] | 1.72 [1.60, 1.86] |
| log(tau) ~ log(BL TSPO) [HC] | 1.48 [1.44, 1.52] | 1.48 [1.44, 1.52] | 1.48 [1.44, 1.52] | 1.48 [1.44, 1.52] |
| TSPO change ~ log(BL TSPO) + log(tau): tau effect [ADS] | 1.08 [1.06, 1.11] | 1.08 [1.05, 1.10] | 1.08 [1.06, 1.11] | 1.08 [1.06, 1.10] |
| TSPO change ~ log(BL TSPO) + log(tau): tau effect [HC] | 1.25 [1.19, 1.32] | 1.25 [1.19, 1.32] | 1.25 [1.19, 1.32] | 1.25 [1.19, 1.32] |
| log(tau) ~ FC to BL-TSPO epicenters [ADS] | 1.50 [1.34, 1.68] | 1.50 [1.34, 1.68] | 1.50 [1.34, 1.68] | 1.50 [1.34, 1.68] |
| log(tau) ~ FC to BL-TSPO epicenters [HC] | 1.14 [1.10, 1.19] | 1.14 [1.10, 1.19] | 1.14 [1.10, 1.19] | 1.14 [1.10, 1.19] |
| log(tau) ~ FC to TSPO-change epicenters [ADS] | 0.74 [0.67, 0.83] | 0.74 [0.67, 0.83] | 0.74 [0.67, 0.83] | 0.74 [0.67, 0.83] |
| log(tau) ~ FC to TSPO-change epicenters [HC] | 1.01 [0.97, 1.05] | 1.01 [0.97, 1.05] | 1.01 [0.97, 1.05] | 1.01 [0.97, 1.05] |
| log(tau) ~ FC-BL + FC-change: FC-BL effect [ADS] | 1.39 [1.22, 1.58] | 1.39 [1.22, 1.58] | 1.39 [1.22, 1.58] | 1.39 [1.22, 1.58] |
| log(tau) ~ FC-BL + FC-change: FC-change effect [ADS] | 0.86 [0.76, 0.97] | 0.86 [0.76, 0.98] | 0.86 [0.76, 0.98] | 0.86 [0.76, 0.98] |
| log(tau) ~ FC-BL + FC-change: FC-BL effect [HC] | 1.14 [1.10, 1.19] | 1.14 [1.10, 1.19] | 1.14 [1.10, 1.19] | 1.14 [1.10, 1.19] |
| log(tau) ~ FC-BL + FC-change: FC-change effect [HC] | 1.01 [0.97, 1.05] | 1.01 [0.97, 1.05] | 1.01 [0.97, 1.05] | 1.01 [0.97, 1.05] |
| log(tau) ~ group × log(BL TSPO): group×BL-TSPO interaction | 0.91 [0.87, 0.95] | 0.91 [0.87, 0.95] | 0.91 [0.87, 0.95] | 0.91 [0.87, 0.95] |
| ΔTSPO ~ log(BL TSPO) + group × log(tau): group×tau interaction | 1.09 [1.05, 1.14] | 1.09 [1.05, 1.14] | 1.09 [1.05, 1.14] | 1.09 [1.05, 1.14] |
| log(tau) ~ group × FC to BL-TSPO epicenters: group×FC-BL interaction | 0.72 [0.66, 0.78] | 0.72 [0.66, 0.78] | 0.72 [0.66, 0.78] | 0.72 [0.66, 0.78] |
| log(tau) ~ group × FC to TSPO-change epicenters: group×FC-change interaction | 1.33 [1.22, 1.45] | 1.33 [1.22, 1.45] | 1.33 [1.22, 1.45] | 1.33 [1.22, 1.45] |

# Table S11: Sensitivity analysis of out-of-sample validation using follow-up fMRI connectivity in all participants with follow-up tau PET

| **Model** | **Test set** | **n** | **Term** | **β [95% CI]** | **exp(β) [95% CI]** | **p** |
| --- | --- | --- | --- | --- | --- | --- |
| Single | ADS | 11 | FC to TSPO BL epicenter | 0.526 [0.41, 0.64] | 1.69 [1.51, 1.90] | <0.001 |
|  | ADS | 11 | FC to TSPO change epicenter | −0.302 [−0.45, −0.16] | 0.74 [0.64, 0.85] | <0.001 |
| Single | HC | 13 | FC to TSPO BL epicenter | 0.061 [0.02, 0.10] | 1.06 [1.02, 1.11] | 0.003 |
|  | HC | 13 | FC to TSPO change epicenter | −0.010 [−0.07, 0.05] | 0.99 [0.93, 1.05] | 0.742 |
| Single | ADS (indep) | 6 | FC to TSPO change epicenter | −0.448 [−0.63, −0.27] | 0.64 [0.53, 0.76] | <0.001 |
| Single | ADS (indep) | 6 | FC to TSPO BL epicenter | 0.555 [0.39, 0.72] | 1.74 [1.48, 2.05] | <0.001 |
| Combined | ADS (indep) | 6 | FC to TSPO BL epicenter | 0.643 [0.46, 0.83] | 1.90 [1.58, 2.29] | <0.001 |
|  |  | 6 | FC to TSPO change epicenter | −0.107 [−0.31, 0.10] | 0.90 [0.73, 1.10] | 0.299 |
| Combined | ADS (all) | 11 | FC to TSPO BL epicenter | 0.576 [0.44, 0.71] | 1.78 [1.56, 2.03] | <0.001 |
|  |  | 11 | FC to TSPO change epicenter | −0.078 [−0.24, 0.08] | 0.93 [0.79, 1.09] | 0.345 |

**Figure S1:** Participant flow diagram. The diagram shows cohort classification, rs6971 exclusions, imaging availability, the complete longitudinal subgroup, the independent epicenter-definition samples, and the primary and sensitivity validation samples. BL, baseline; FU, follow-up; LAB, low-affinity binder; fMRI, functional magnetic resonance imaging.

**Figure S2:** Cortical TSPO and tau epicenter maps. Colored parcels indicate the top 20% of Schaefer-200 cortical regions used as baseline-TSPO, TSPO-change, and the overlap between these, in ADS (a-c) and HC (d-f), displayed in lateral and medial views. Corresponding baseline-TSPO and TSPO-change parcels and Yeo-7 network assignments are listed in Tables S8 and S9.
